# Complexation of Citalopram with β‑Cyclodextrin, Mono-subetadex, and Subetadex: Phase Solubility, Hummel–Dreyer, Affinity Capillary Electrophoresis, ITC, and NMR Studies

**DOI:** 10.1021/acsomega.5c13544

**Published:** 2026-04-15

**Authors:** Dóra V. Ujj, Petr Kasal, Ida Fejős, Szabolcs Béni, József Kardos, Gábor Benkovics, Erika Bálint, Béla Mátravölgyi

**Affiliations:** † Department of Organic Chemistry and Technology, Faculty of Chemical Technology and Biotechnology, Budapest University of Technology and Economics, Műegyetem rkp. 3, Budapest H-1111, Hungary; ‡ Institute of Organic Chemistry and Biochemistry, 89220 Czech Academy of Sciences, Národni 3, Prague 1 CZ-110 00, Czech Republic; § Department of Pharmacognosy, Semmelweis University, Üllői út 26, Budapest H-1085, Hungary; ∥ Center for Pharmacology and Drug Research & Development, Semmelweis University, Üllői út 26, Budapest H-1085, Hungary; ⊥ Integrative Health and Environmental Analysis Research Laboratory, Department of Analytical Chemistry, Institute of Chemistry, ELTE Eötvös Loránd University, Pázmány Péter sétány 1/a, Budapest H-1117, Hungary; # ELTE NAP Neuroimmunology Research Group, Department of Biochemistry, ELTE Eötvös Loránd University, Pázmány Péter sétány 1/C, Budapest H-1117, Hungary

## Abstract

Cyclodextrins (CDs) have a number of important properties, such as chiral separation or the enhancing of water solubility of various substances. In the present study the complexation properties of sugammadex analog CDs, such as subetadex (SBX) and mono-subetadex (monoSBX) were investigated and compared with the native β-CD. The complexation features were investigated at pH 7.4 using citalopram, a selective serotonin reuptake inhibitor antidepressant, as a model compound. The stability constants were investigated by phase-solubility test, the Hummel–Dreyer (HD) method, affinity capillary electrophoresis, and ITC and the different analytical techniques were comprehensively evaluated and compared. The NMR studies supported the results from the investigated techniques and the complex structure was characterized by ROESY NMR studies. The phase-solubility study yielded results that deviated from the other three techniques, with monoSBX showing the highest stability constant. In the case of the HD method, the value of stability constants significantly differed from ACE and ITC, but the trend of CD stability followed the same order (β-CD < monoSBX < SBX). The ACE and ITC methods gave very similar results for β-CD and monoSBX, with some deviations for SBX, although still within the same order of magnitude. Based on the results ACE and ITC appear to be the most reliable techniques for determining CD-guest stability constants.

## Graphical abstract

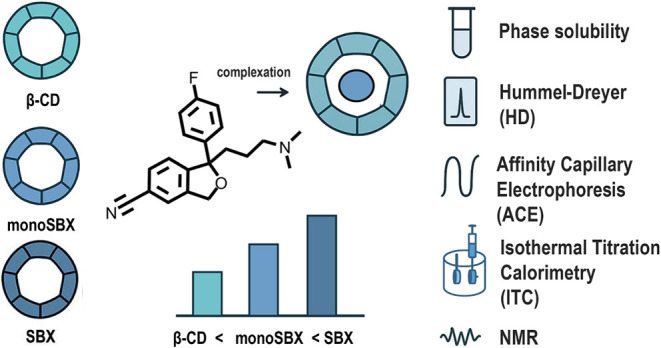

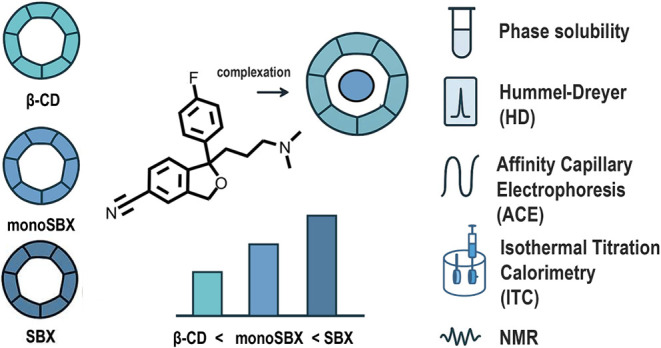

## 1 Introduction

The importance of cyclodextrins (CDs) is indisputable in many fields of the industry. In particular, the demand for diversely substituted CDs has increased in the pharmaceutical, cosmetic, and food industries. They can be used to enhance the water solubility of a guest molecule,^1^ to increase its stability, or to achieve chiral separation. As recently highlighted by Ghitman et al., cyclodextrins have been widely explored as drug delivery systems.^2^ The reason for their widespread use originates from their outstanding encapsulation properties. To characterize the complex formed between the CD and the guest molecule, the association constant, also known as the complex stability constant (*K*_A_) is used. There are several techniques to determine *K*_A_: spectroscopic, calorimetric, electroanalytical, chromatographic, and polarimetric techniques. Spectroscopic techniques, in particular NMR, are essential for the characterization of the structure of the formed inclusion complexes.^3^ However, for example, electroanalytical techniques or HPLC, affinity capillary electrophoresis (ACE), and isothermal titration calorimetry (ITC) techniques can be of great help in understanding the thermodynamics, stoichiometry, kinetics, or other aspects of the complex formation.^3^ A very detailed study was written by Singh et al. in 2010, which describes and compares the techniques used to determine the stability constants.^4^

In a recent publication of Puskás et al., several examples were collected for the use of native and randomly substituted CDs in the pharmaceutical industry.^5^ Randomly substituted CDs are mixtures of a large number of isomers and derivatives with different degrees of substitution. While their synthesis is easier, their analytical characterization and pharmaceutical licensing are more difficult. These CDs are usually characterized only by the average degree of substitution (DS), which is not sufficient, since this value does not provide any information on the substitution pattern.^6^ The properties of these CDs need to be very thoroughly investigated, which is not an easy task, and even their degradation products can be extremely diverse, almost impossible to characterize, which causes the difficulties when it comes to licensing.

Compared to randomly substituted CDs, the analysis of single isomer (SI) CDs is more straightforward. Among SI CDs, monosubstituted (only one substituent on a CD molecule in a determined position) and persubstituted (all hydroxyl groups substituted in the same positions) CDs are the most common. Kasal and Jindřich^7^ recently reviewed the most useful and well-described methods for the preparation of mono-6-substituted CD derivatives.

In addition to monosubstituted CDs, there are several well-established techniques for the synthesis of persubstituted CDs, and they can be implemented, among others, in batch reaction, in a ball mill, or in a microwave reactor as well.^8–10^ The most prominent of these CDs is sugammadex (Bridion). It has very high affinity toward curare analogs, especially rocuronium (complex stability constant is app. 1.8 × 10^7^ M^–1^), which is widely used in clinical practice.^11^ The use of SI CDs (either mono- or persubstituted) is widespread, for example, in chiral analysis, as they allow a more precise determination of the stability constant and therefore a predictable separation.^6,12,13^

Subetadex (SBX, for the schematic structure, see Figure 1) is a structurally defined β-CD, persubstituted at C-6 by 3-mercaptopropionic acid. SBX shows a high structural similarity to sugammadex, differing only in the number of glucose units. Previous studies have demonstrated that SBX has significant solubility-enhancing and enantiomer recognition properties, but these features are pH-dependent. The chiral separation predominates under acidic condition, while the complex formation is dominant under neutral condition.^14^ Mono-subetadex (monoSBX, for the schematic structure, see Figure 1) is a monosubstituted β-CD containing only one 3-mercaptopropionic acid side chain at position C-6. The protonation properties of SBX and its homologues (sualfadex and sugammadex) have already been investigated by Kalydi et al.^15^ There are also examples for the use of these CDs in ACE.^14,16,17^

**Figure 1:**
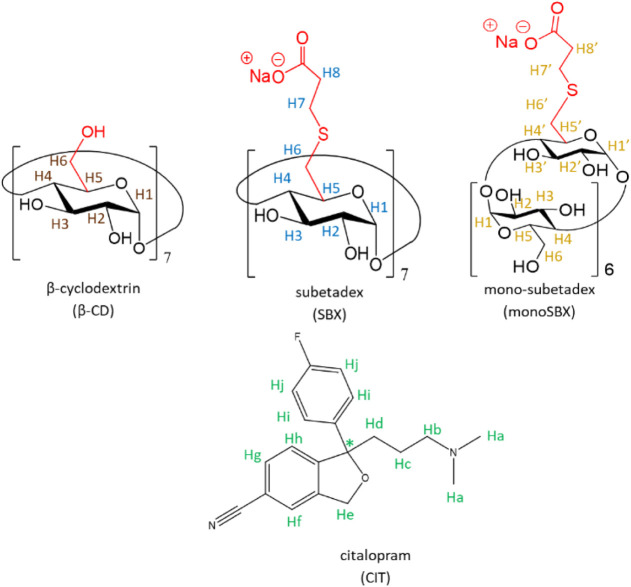
Schematic structure of the studied CDs and the chemical structure of citalopram.

To compare the complexation behavior of various CDs, citalopram (CIT) was chosen as a model compound (Figure 1). CIT is a selective serotonin reuptake inhibitor (SSRI), frequently administered in the treatment of major depressive disorders. It contains a chiral center, but both racemic and enantiomerically pure versions are used for therapeutic purposes. The separation of the enantiomers of CIT is also a topic of great relevance.^18^ However, CIT is interesting not only from the point of view of enantiomer separation. Although it is a second-generation antidepressant, CIT has several side effects, such as cardiovascular, neurological, and gastrointestinal side effects, seizures, sexual dysfunction, and weight gain.^19,20^ CDs can improve the chemical stability of the active ingredient^21^ and can reduce or mask the side effects and local irritations of the drugs.^22–24^ Furthermore, CDs may increase their water solubility, which may lead to an increased drug efficacy. Therefore, lower doses can be administered, resulting in less toxicity in the body.^25^

The CIT–native β-CD complex has been investigated in the literature in various ways, such as NMR, HPLC, and X-ray^26–28^ furthermore CIT and its analogs have also been studied by ACE with a number of CDs.^12,29^ However, to the best of our knowledge, the complex of CIT and sugammadex analog CDs has not been investigated up to now, especially not with different techniques.

The scope of this paper is to determine and compare the stability constants obtained by using different but complementary techniques and to investigate the effect of the substituents on the stability constant. Furthermore, we aimed to investigate the 3D structures of the complexes by NMR. From these results, we aimed to draw conclusions about which techniques might be the most advantageous among the tested methods.

## 2 Methods and Materials

### 2.1 Materials

The fine chemical grade β-CD used for the synthesis of SBX and monoSBX was sourced from CycloLab Ltd. (Budapest, Hungary). Reagents such as triphenylphosphine (PPh_3_), iodine, sodium methoxide (NaOMe), sodium hydroxide, 3-mercaptopropionic acid, *p*-toluenesulfonyl chloride were sourced from TCI (Tokyo, Japan). Solvents such as *N*,*N*-dimethylformamide (DMF), dimethyl sulfoxide (DMSO), methanol, acetone, dioxane, propan-1-ol (*n*-PrOH), sulfuric acid (H_2_SO_4_), ethyl acetate, hydrogen chloride solution (HCl), and ammonium hydroxide solution (NH_3_ aq solution) were of reagent grade and sourced from Reanal Ltd. (Budapest, Hungary).

Analytical measurement for the determination of the complex stability constants: β-CD (fine chemical grade) was sourced from CycloLab Ltd. All chemicals that were used for the phase solubility, ACE, Hummel–Dreyer method, ITC, and NMR study were of analytical grade. Sodium dihydrogen phosphate (NaH_2_PO_4_), DMSO, sodium hydroxide (NaOH), diethylamine (DEA), and deuterium oxide (D_2_O, 99.9% D) were purchased from Sigma-Aldrich, Merck KGaA (St. Louis, MO, USA), acetonitrile (ACN), and methanol (MeOH) were HPLC grade and purchased from VWR (Darmstadt, Germany). Bidistilled Millipore water (PW) was used. Citalopram (racemic) and S-citalopram oxalate were purchased from TCI (Tokyo, Japan).

### 2.2 Synthesis of SBX and monoSBX

SBX and monoSBX were synthesized in our laboratory at the Budapest University of Technology and Economics. SBX was synthesized from per­(6-deoxy-6-iodo)-β-CD and monoSBX was synthesized from 6^A^-*O*-*p*-toluenesulfonyl-β-CD. The per­(6-deoxy-6-iodo)-β-CD was synthesized as previously described by Ashton et al.^30^ and the 6^A^-*O*-*p*-toluenesulfonyl-β-CD was synthesized as previously described by Popr et al.^31^ The synthesis of monoSBX and SBX was in a similar way as previously described.^14^ The syntheses of per­(6-deoxy-6-iodo)-β-CD, 6^A^-*O*-*p*-toluenesulfonyl-β-CD, monoSBX, and SBX are detailed in the Supporting Information.

### 2.3 Phase Solubility Study

Phase solubility measurements were performed according to Higuchi and Connors.^32^ The concentration of β-CD, monoSBX, and SBX was in the range of 0–5 mM in 30 mM phosphate buffer (preparation of the buffer: 30 mM NaH_2_PO_4_ solutions were prepared and the pH was adjusted to 7.4 with 1 M NaOH). In all cases, CIT was added in excess to the CD-containing solutions, resulting in a suspension. The suspensions were stirred with a magnetic stirrer at 750 rpm at 25 °C for 24 h and filtered using 0.45 μm FilterBio PTFE Syringe filters (FilterBio Membrane Co., Nantong, China).

The CIT concentration in the filtered samples was determined with an Agilent 1100 HPLC system equipped with a photodiode array detector and a Lux Cellulose-1 250 mm × 4.6 mm, 5 μm (Phenomenex Inc., Torrance, CA, USA) analytical column. The following HPLC method was used: mobile phase: ACN:PW = 60:40 + 0.1% DEA, flow rate: 0.8 mL/min, injection volume: 10 μL, column temperature: 25 °C, and detection at 222 nm. A successful separation of the two enantiomers was achieved, and no interfering peaks were detected. For more detailed parameters and chromatograms, please see the Supporting Information.

The phase solubility curves were plotted; the stability constant (*K*_A_) and the complexation efficacy (CE) were calculated using eqs 1 and 2:

1 $$K_{A}=\frac{\mathrm{slope}}{S_{0}\left(1-\mathrm{slope}\right)}$$

2 $$\mathrm{CE}=\frac{\mathrm{slope}}{1-\mathrm{slope}}$$

where *S*_0_ is the solubility of a poorly soluble guest molecule, without CD. It is worth noting that the solubility of the guest molecule strongly influences the value of *K*_A_, since the solubility without CD is included in the stability constant equation.

### 2.4 Hummel–Dreyer Method by HPLC

The association constants were also determined by the Hummel–Dreyer (HD) method.^33,34^ According to the method, the eluent contained the guest molecule (which was CIT in our case). The CD samples are dissolved in the eluent and injected to the column. When complex formation occurs, a positive peak and then a negative peak can be observed in the chromatogram. The retention time of the negative peak is equal to the retention time of the free CIT molecule, and the area of the negative peak is proportional to the concentration of the complex. A calibration curve from the CIT standard is used to determine the concentration proportional to the area of the negative peak. From the calculated concentration, the stability constant can be determined using eq 3:

3 $$K_{A}=\frac{Q_{S-CD}}{\left[S\right]\cdot \left(Q_{CD}-Q_{S-CD}\right)}$$

where *Q*_S–CD_ = total amount of the complex (dimension: M); *Q*_CD_ = total amount of CD applied (dimension: M); [*S*] = concentration of the guest molecule in the eluent (dimension: M).

The stability constants were measured in a buffer of 30 mM phosphate, pH 7.4. The following method was used for the HPLC measurement: column: YMC-Pack C4 (100 mm × 2.1 mm, 5 μm, 30 nm), isocratic elution: ACN:30 mM phosphate buffer (pH 7.4) = 10:90 + 0.03 mM CIT, flow rate: 1.2 mL/min, column temperature: 45 °C, injection: 10 μL, detection at 222 nm. Preparation of the eluent: 30 mM NaH_2_PO_4_ was dissolved in the appropriate volume of PW, the pH was adjusted to 7.4 with 1 M NaOH and the appropriate volume of ACN was added to the eluent and finally the appropriate amount of CIT was dissolved in the eluent. For more detailed parameters and chromatograms, please see the Supporting Information.

### 2.5 Affinity Capillary Electrophoresis Conditions and Sample Preparation

In the experiments, a fully automated HP^3D^CE instrument (Agilent Technologies, Waldbronn, Germany) equipped with a photodiode array detector was applied. The measurements were performed on untreated fused silica capillaries (Agilent Technologies, Waldbronn, Germany) with a total length of 48.5 cm, an effective length of 40 cm, and an inner diameter of 50 μm. The voltage was set to +15 kV and the detection was performed via on-column measurements of the UV absorption at 200 nm. The capillary temperature during the operation was set to 25 °C. The capillary was rinsed with 1 M NaOH (30 min), 0.1 M NaOH (30 min), and PW (30 min) every day, prior to the start of the measurements. Before the runs, the capillary was flushed with background electrolyte (BGE) (2 min). BGE solutions were prepared as follows: 30 mM NaH_2_PO_4_ solutions were prepared, and the pH was adjusted to 7.4 with 1 M NaOH. The solutions were filtered and stored at 12 °C before use. Prior to the application, the appropriate amount of CD (β-CD: 0.5–10 mM; monoSBX: 0.05–5 mM; SBX: 0.05–5 mM) was dissolved in the BGE, filtered again, and the pH of the resulting solution was checked. During the preparation of the negatively charged CD-containing buffer the ionic strength was always constant. A stock CIT solution of 1 mg/mL concentration was prepared from the solid samples with 100% MeOH, and right before the measurements 25-fold dilutions with PW were used to prepare the sample solution applying 0.1% DMSO as an electroosmotic flow (EOF) marker. Samples were injected hydrodynamically by applying a pressure of 150 mbar·s. Each experiment was run in triplicate.

The stability constants were determined using the CEval software.^35^ In order to determine the complex stability constants, the first step is to determine the effective mobility of the analyte at specific CD concentrations. Triangular peaks caused by electromigration dispersion are often observed in the ACE measurements. To suppress these errors, the Haarhof–Van der Linde (HVL) function (eq 4) can be used to determine the effective mobility more accurately:

4 $$HVL_{\delta }\left(t;{\;a}_{0},a_{1},a_{2},a_{3\delta }\right)=\frac{\frac{a_{0}}{a_{2}a_{3\delta }\sqrt{2\pi }}\exp\left[-\frac{1}{2}{\left(\frac{t-a_{1}}{a_{2}}\right)}^{2}\right]}{\frac{1}{\exp\left(a_{3\delta }\right)-1}+\frac{1}{2}\left[1+\mathrm{erf}\left(\frac{t-a_{1}}{\sqrt{2a_{2}}}\right)\right]}$$

where *a*_0_ is the area of the HVL function *a*_1_ is the position of the Gaussian component corresponding to the migration time of the analyte, *a*_2_ is the standard deviation of the Gaussian component, and *a*_3δ_ is the triangular distortion.

Assuming a 1:1 complexation ratio (based on the ITC and NMR results) and that the equilibrium CD concentration is almost equal to the total CD concentration, a relationship between the effective mobility relative to the EOF and the complex stability constant can be described by eq 5:

5 $$\mu_{eff}=\frac{\mu_{A}+\mu_{ACD}K_{A}\left[CD\right]}{1+K_{A}\left[CD\right]}$$

where μ_eff_ is the effective mobility of the analyte, μ_A_ and μ_ACD_ are the free and complexed analyte mobilities, [CD] is the concentration of the selector, and *K*_A_ is the complex stability constant.

To ensure precise and robust determination of complex stability constants, the results were corrected with ionic strength^36^ and viscosity.^37^

### 2.6 Isothermal Titration Calorimetry (ITC) Study

ITC experiments were performed on a MicroCal PEAQ-ITC instrument (Malvern Panalytical, Worcestershire, UK). 1 mM CIT was dissolved in 30 mM phosphate buffer, pH 7.4 (30 mM NaH_2_PO_4_ solutions were prepared, and the pH was adjusted to 7.4 with 1 M NaOH). β-CD, monoSBX, and SBX were dissolved in the same buffer at 10 mM, 5.6 mM, and 20 mM concentrations, respectively. Solutions were filtered through a 0.22 μm PVDF syringe filter. 0.5 or 1 mM CIT solutions were filled in the cell and titrated with the CDs applying 18 or 36 titrations of 2 or 1 μL aliquots, respectively. The spacing between injections was 300 s, and a reference heat of 5 μcal/s was used. Control measurements were carried out by injecting CDs to the phosphate buffer solution with no CIT in the cell. The enthalpy changes of the control measurements were subtracted from the CD-to-CIT titrations. The results were evaluated using MicroCal PEAQ-ITC Analysis Software (Malvern Panalytical).

### 2.7 NMR Study of CIT–CD Interactions

NMR experiments were performed on a Bruker Avance Neo 400 MHz, a Bruker Avance III 500 MHz spectrometer and a Bruker Avance III 700 MHz instrument equipped with a Prodigy TCI H&F–C/N–D z-gradient probe head using the built-in pulse sequences of Topspin. The probe temperature was maintained at 298 K, and standard 5 mm NMR tubes were used.

All samples were prepared under conditions identical with those used in the phase solubility studies, except that only one CD concentration was applied. For β-CD, monoSBX, and SBX, 3 mM samples were prepared in 30 mM NaH_2_PO_4_ (D_2_O), with the pH adjusted to 7.4 using 1 M NaOD. The pH was confirmed after complete dissolution. Excess CIT was added to each sample, and the suspensions were stirred with a magnetic stirrer at 25 °C. After 24 h, the samples were filtered using 0.45 μm FilterBio PTFE syringe filters (FilterBio Membrane Co., Nantong, China) and transferred into an NMR tube. Based on the phase solubility curves, the CD:CIT ratio in these samples was 1:1.

During the NMR measurements, separation of the CIT enantiomers was observed (as diastereomeric splitting). Therefore, CIT-β-CD (ratio: 1:15) was spiked with enantiomerically pure S-CIT oxalate to enable the unambiguous assignment of the individual CIT enantiomer signals. The assignment is shown in Section 6.2 of the Supporting Information.

The stoichiometry of CIT complexation with β-CD and SBX was investigated by the Job’s method^38^ of continuous variation. Samples were prepared in 30 mM NaH_2_PO_4_ (D_2_O:PW = 10:90), with the pH adjusted to 7.4. The total molar concentration of the two components, CIT + CD, was kept constant at 1 mM, while the mole fraction of CIT was varied from 0.1 to 1 in increments of 0.1.

The ^1^H NMR chemical shifts of selected, well-resolved characteristic CIT resonances were monitored, and the complexation-induced chemical shift changes were calculated relative to the corresponding resonances measured in the absence of CD. For the construction of Job plots, the shift differences were multiplied by the mole fraction of the CIT and plotted against the mole fraction. The illustrative spectra are collected in Section 6.3 of Supporting Information.

In the NMR titration experiments, the concentration of CIT was kept constant at 0.5 mM. The concentration of β-CD was varied between 0 and 12.5 mM (0.0, 0.05, 0.25, 0.5, 1.0, 2.5, 5.0, 7.5, 10.0, and 12.5 mM), while the concentration of SBX was adjusted between 0 and 16.7 mM (0.0, 0.05, 0.25, 0.5, 1.0, 2.5, 5.0, 7.5, 10.0, 12.5, and 16.7 mM). All samples were prepared in 30 mM NaH_2_PO_4_ (D_2_O: PW = 10:90), with the pH adjusted to 7.4. The ^1^H NMR chemical shifts of selected, well-resolved characteristic resonances of CIT were monitored and plotted to construct the Scott plot diagram. The concentration of CD is shown in mM on the *x*-axis, while the concentration of CD divided by the chemical shift change of the selected proton of CIT is shown in mM/ppm on the *y*-axis. The illustrative ^1^H NMR spectra and Scott plot^39^ diagrams are in Section 6.4 of the Supporting Information.

To further validate the 2D ROESY NMR results, 1D selective ROESY experiments were performed for the CIT−β-CD and CIT–SBX complexes. In these experiments, selected resonances of either the CIT or the CD were selectively irradiated, resulting in negative-phase signals for the irradiated protons. Protons exhibiting spatial proximity to the irradiated resonance appeared as positive-phase signals in the spectrum. For these measurements 3 mM β-CD or SBX solutions were prepared in 30 mM NaH_2_PO_4_ (D_2_O), with the pH adjusted to 7.4 using 1 M NaOD. The pH was verified after complete dissolution. Excess CIT was then added to each sample, and the suspensions were stirred with a magnetic stirrer at 25 °C for 24 h. After equilibration, the samples were filtered through a 0.45 μm FilterBio PTFE syringe filter (FilterBio Membrane Co., Nantong, China) and transferred into NMR tubes for analysis. The illustrative spectra are collected in Section 6.5 of the Supporting Information.

## 3 Results and Discussion

### 3.1 Phase Solubility Study

The phase solubility analysis is a widely used method for evaluating the effect of CD on the complexation and solubility of guest molecules. However, this method is neither particularly fast nor efficient in terms of tools or materials. Several milligrams of CD and guest molecule are needed for the preparation of the samples, and the associated HPLC analysis is also very time-consuming and demands significant amounts of eluent. Despite these limitations, phase solubility analysis provides a wealth of information including the extent of solubility enhancement of the guest molecule, the stability constant, and the guest-to-host ratio within the complex.

In the phase solubility study, β-CD, monoSBX, and SBX were tested. For better comparability with ACE measurements, the tests were performed in 30 mM phosphate buffer at pH 7.4. The phase solubility curves are shown in Figure 2, and the results are collected in Table 1.

**Figure 2:**
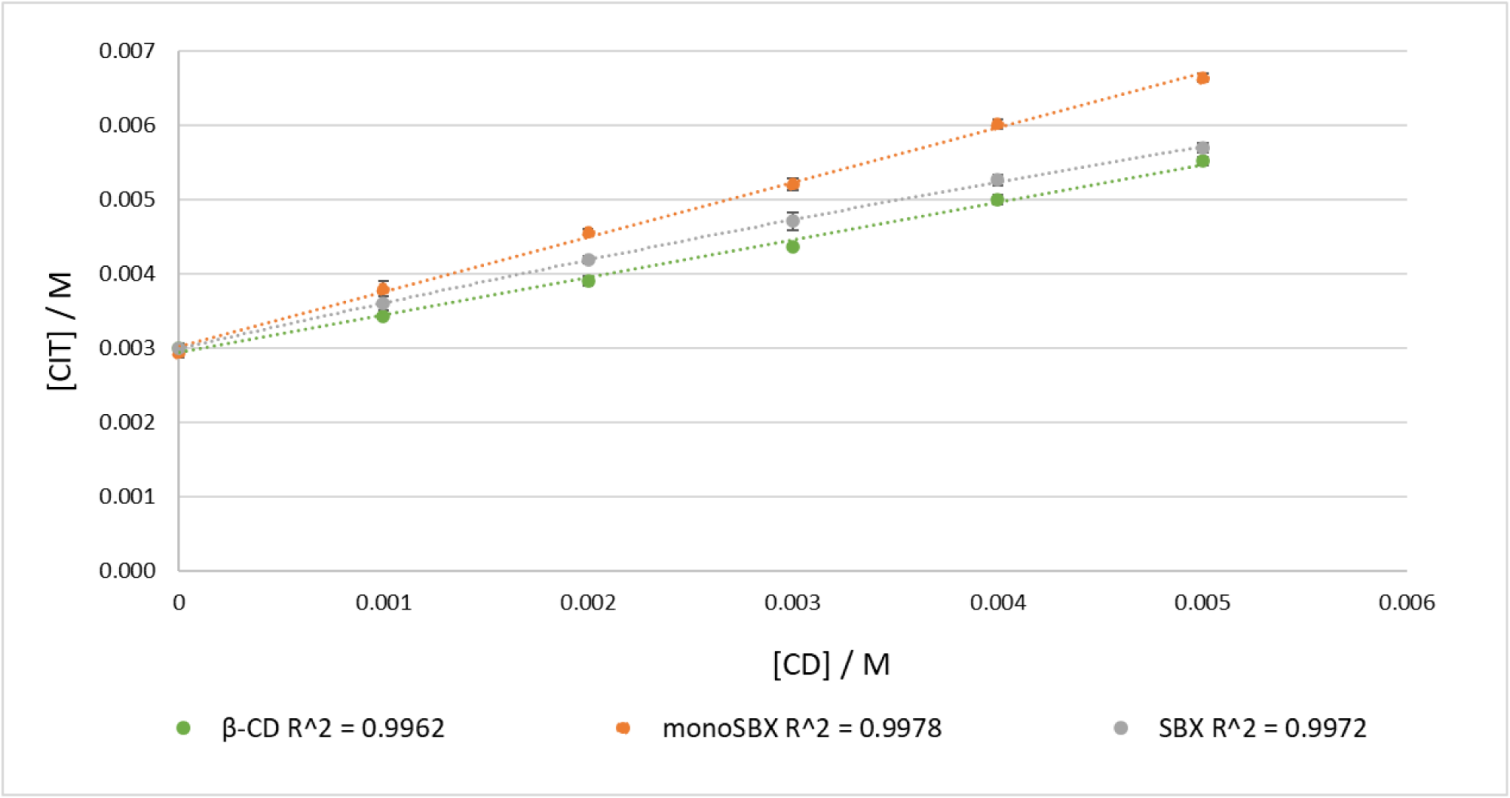
Phase solubility diagram of CIT with β-CD, monoSBX, and SBX in 30 mM phosphate buffer pH 7.4, at 25 °C, after 24 h obtained by the HPLC quantification method (ACN:PW = 60:40 + 0.1% DEA, flow rate: 0.8 mL/min, injection volume: 10 μL, column temperature: 25 °C, detection at 222 nm).

**Table 1:** Stability Constants (*K*_A_), Complexation Efficiencies (CE), and Solubility Enhancement (*S*_MAX_/S_0_) for Complexes of CIT with the Tested CDs in Water at 25 °C, after 24 h (Number of Measurements = 3; SE = Standard Error of the Mean)

	β-CD	monoSBX	SBX
*S*_0_/(M)	0.0029	0.0029	0.0029
Slope	0.487	0.738	0.554
*K*_A_ /(M^–1^)	320 ± 4	950 ± 10	430 ± 30
CE/(−)	1.00 ± 0.04	2.81 ± 0.03	1.25 ± 0.10
*S*_MAX_/*S*_0_	1.8 ± 0.1	2.3 ± 0.1	1.9 ± 0.1

The *K*_A_ value of the complexes indicates the binding strength between CD and the guest molecule, but it is important to note that various types of complexes may coexist in the solution including inclusion and noninclusion complexes, as well as aggregates of CDs or CD–guest complexes. For example, aggregates can solubilize guest molecules through a micellar-like mechanism. Thus, the stability constants determined from phase solubility profiles primarily reflect aggregate-assisted solubilization rather than the formation of true inclusion complexes. CE offers a more precise method for evaluating the solubilizing effects of cyclodextrins.^40^

The solubility of CIT increased linearly as a function of the concentration of CDs resulting in an A_L_ type profile, which indicates a 1:1 host:guest complex formation.^32^ The data show that β-CD had the least effect on enhancing the solubility of CIT, while monoSBX demonstrated the highest solubility enhancement among the tested CDs. These trends are consistent with the *K*_A_ and CE values.

Notably, the behavior of the CIT–SBX complex represents an important observation: although the degree of substitution of SBX would suggest the formation of the most stable complex with CIT, the solubility of CD–guest complexes does not necessarily increase and may even decrease in certain cases, potentially leading to complex precipitation.^32^ Since the results obtained were unusual, the CD–CIT complexes were examined using additional analytical techniques: HD method, ACE, and ITC.

The HPLC measurements were carried out on a chiral column to investigate the ratio of the citalopram enantiomers. If a CD solubilizes CIT in an enantioselective manner, the ratio of the two enantiomers would deviate from 1:1. However, the peak ratio of the two CIT enantiomers remained 1:1, indicating that no chiral recognition occurred in either water or 30 mM phosphate buffer with β-CD, monoSBX, or SBX. Illustrative chromatograms are shown in Supporting Information Figure S3.

### 3.2 Hummel–Dreyer Method by HPLC

The HD method is a reliable HPLC-based technique for analyzing the strength of noncovalent interactions of inclusion complexes and thus the complex stability. This method is fast, requires only a small amount of guest and CD, but has a high eluent consumption, making it less environmentally friendly.

The chromatograms of CIT and its CD complexes are shown in Figure 3. The upper chromatogram (**A**) was obtained by injecting a 0.3 mM excess of the CIT (calibration sample) onto the column with calibration performed in the range of 0.03–0.63 mM. The lower chromatogram (**B**) was recorded after the injecting 3 mM β-CD (blue), 3 mM monoSBX (green), and 3 mM SBX (red), all dissolved in the mobile phase. Positive peaks appear at *t*_0_, due to the lack of retention for the tested CDs on the C4 column.

**Figure 3:**
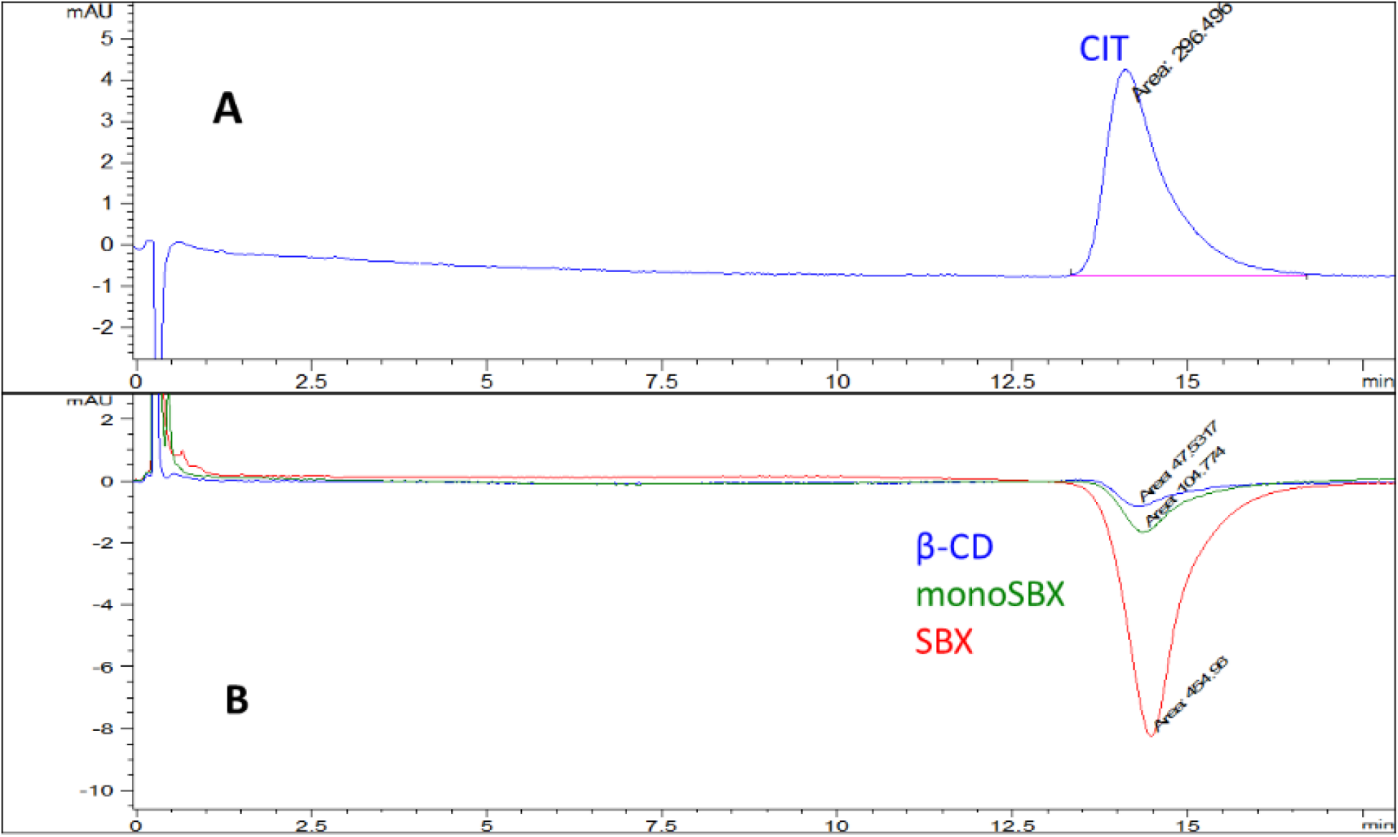
**A**: Chromatogram of reference CIT, **B**: Comparative chromatograms of CIT−β-CD/monoSBX/SBX complexes, measured by HPLC (YMC-Pack C4 (100 × 2.1 mm, 5 μm, 30 nm), isocratic elution: ACN:30 mM phosphate buffer (pH 7.4) = 10:90 + 0.03 mM CIT, flow: 1.2 mL/min, column temperature: 45 °C, injection: 10 μL, detection at 222 nm).

The calculated *K*_A_ values for the complexes for CIT–CD complexes in 30 mM phosphate buffer are the followings: CIT- β-CD: 475 ± 1 M^–1^; CIT-monoSBX: 760 ± 5 M^–1^; CIT-SBX: 3397 ± 14 M^–1^. These values differ from those obtained via the phase solubility test, likely due to variations in experimental conditions, such as temperature. Notably, the ranking of CDs by stability constant also differs, with the highest *K*_A_ observed for the CIT–SBX complex. This supports the hypothesis that the stability constant derived from the phase solubility study for SBX should be interpreted with caution. According to the HD method, the stability increases with the number of side chains on the CD (β-CD < monoSBX < SBX).

### 3.3 Affinity Capillary Electrophoresis Study

The stability constants between CDs and guest molecules can be determined by the environment-friendly, simple, fast, low-cost, and low-sample-consuming ACE method. This study examined the complexation of CIT with β-CD, monoSBX, and SBX to assess the impact of the number of side chains and to compare the ACE results with those from the phase solubility test and the HD method.

ACE is a widely used technique for analyzing charged compounds, making BGE buffer pH selection a critical aspect of method development. ACE operates on the principle that complexation alters the charge-to-size ratio and, consequently, the electrophoretic mobility of the analyte. Under acidic and neutral conditions, CIT carries a positive charge (p*K*_*a*_ 9.5),^41^ allowing the neutral β-CD to be included in the study. The monoSBX and SBX are deprotonated under neutral and slightly alkaline conditions, ensuring good water solubility. A 30 mM phosphate buffer (pH 7.4) was selected to mimic physiological conditions while providing a sufficient buffer capacity. To prevent excessive current and concomitant Joule heating, the applied voltage was set to 15 kV. Charge plays a crucial role in ACE. At pH 7.4, monoSBX carries one negative charge, while SBX carries seven, leading to significant changes in the CIT migration time at low SBX concentrations. The effect of lower SBX concentrations (0.05–0.25 mM) was also examined, revealing that 0.05 mM SBX in the buffer caused CIT to migrate as a negatively charged complex. Overlaid electropherograms are shown in Figure 4 for various CD concentrations in the case of native β-CD, monoSBX, and SBX. Further electropherograms along with the fitted nonlinear curves are given in Supporting Information Figures S6 and S7.

**Figure 4:**
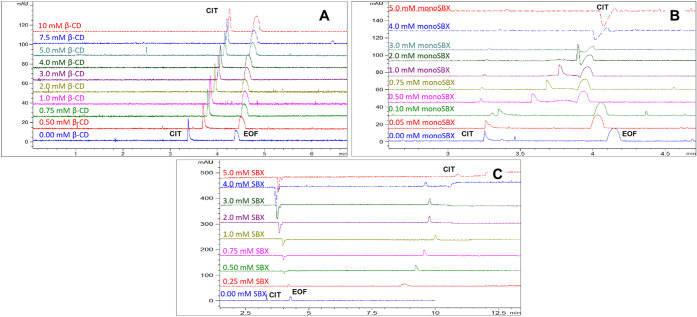
Overlaid electropherograms of CIT applying various concentrations of (A) β-CD, (B) monoSBX, and (C) SBX in 30 mM phosphate buffer, pH 7.4 (Conditions: 50 μm untreated fused silica capillary, 48.5 cm/40 cm; 25 °C; +15 kV; 200 nm).

The overlaid electropherograms demonstrate that, when CIT forms a complex with the noncharged β-CD, the total charge remains positive, causing it to migrate before the EOF. In the CIT–monoSBX complex, increasing the CD concentration in the BGE gradually neutralizes the total charge, resulting in migration near the EOF. In contrast, the CIT–SBX complex carries a significant negative charge migrating in the negative region of the electropherogram (please see the demonstrative overlaid electropherograms in Figure 4) CIT migrated as a single peak without any resolution with all three CDs, thus chiral recognition could not be observed under the studied conditions.

To characterize the complex formation of CIT with the 3 CDs, complex stability constant values were determined by ACE using nonlinear curve fitting. The calculated stability constants are as follows: CIT−β-CD: 1890 ± 10 M^–1^; CIT–monoSBX: 2300 ± 100 M^–1^; CIT–SBX: 38600 ± 400 M^–1^. The order of stability constants (β-CD < monoSBX < SBX) aligns with the results from the HD method, though ACE yielded consistently higher *K*_A_ values. Furthermore, the CIT–SBX complex exhibited significantly greater stability than those with β-CD or monoSBX, highlighting the crucial role of the anionic side chain in complex stability.

### 3.4 Isothermal Titration Calorimetry

ITC is a widely used technique to investigate the binding affinity of biomolecules. Upon injection of the titrant into the guest solution in the calorimeter cell, the heat of the binding reaction is measured at constant temperature and pressure. The method is capable of determining the thermodynamic parameters of complex formation, such as the binding enthalpy changes (Δ*H*), stability constant (*K*_A_) in the range of 10^2^–10^8^ M^–1^, the entropy changes (Δ*S*), and the stoichiometry of complex formation.^42^ ITC has been used to study the inclusion complex formation of CDs with different small molecules.^43,44^ In the present work, β-CD, monoSBX, and SBX solutions were injected into the CIT solution in the calorimeter cell in a buffer of 30 mM phosphate, pH 7.4 at 25 °C, reaching a final 2–3-fold molar excess, and the reaction heats were measured (Figure 5, Supporting Information Figures S8–S10). Fittings to the normalized enthalpy changes provided the inclusion complex stability constants of 1836 ± 99 M^–1^, 2293 ± 257 M^–1^, and 18135 ± 441 M^–1^, for β-CD, monoSBX, and SBX with CIT, respectively (Table 2, Supporting Information Table S3). For β-CD and monoSBX, these values are similar within experimental error to the results of ACE experiments. SBX exhibited the highest affinity for CIT; however, the stability constant determined by ITC is about half of that by ACE (38600 ± 400 M^–1^). This deviation could be attributed to ACE measurements being considerably more influenced by the charge states of the analytes than ITC measurements. The complex formation was enthalpy-driven for all CDs with a small favorable entropy contribution in the case of β-CD and small unfavorable entropy changes for monoSBX and SBX (Table 2). The stoichiometry provided by the fitting was consistent with 1:1 complex formation.

**Figure 5:**
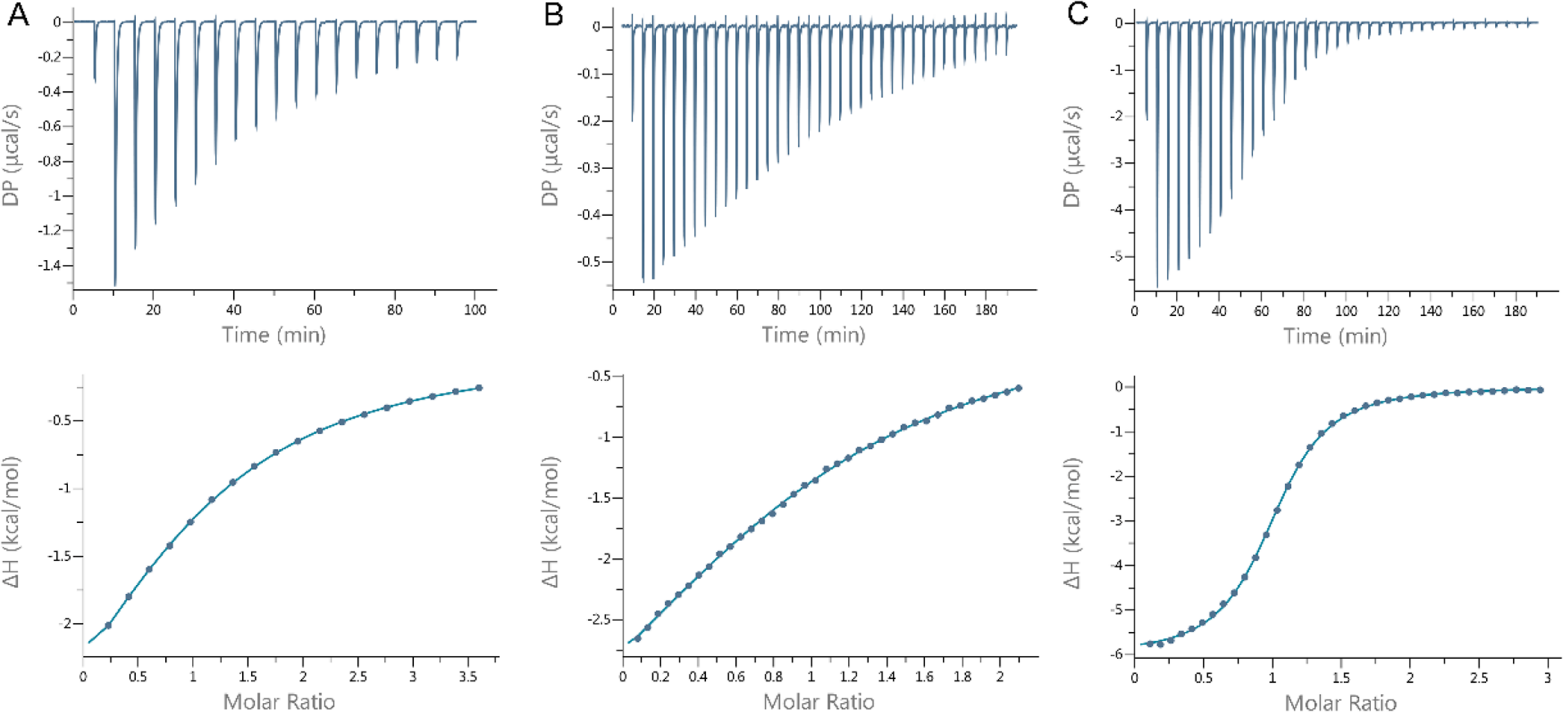
ITC study of the complex formation of CIT with CDs. 0.5 mM CIT was titrated with 2 μL aliquots of 10 mM β-CD (A) and 1 μL aliquots of 5.6 mM monoSBX (B). 1 mM CIT was titrated with 1 μL aliquots of 20 mM SBX (C). Experiments were carried out in 30 mM phosphate buffer, pH 7.4, at 25 °C. Titration heating profiles and the normalized injection enthalpy changes after CD → buffer control subtraction are presented at the top and bottom panels, respectively.

**Table 2:** Thermodynamic Parameters of CIT–CD Complexation Determined by ITC^a^

	*K*_D_ (μM)	*K*_A_ (M^–1^)	Δ*H* (kcal/mol)	Δ*G* (kcal/mol)	–*T*ΔS (kcal/mol)
β-CD	545 ± 26	1836 ± 99	–4.36 ± 0.17	–4.46	–0.10
monoSBX	437 ± 41	2293 ± 257	–4.91 ± 0.34	–4.59	0.32
SBX	55.2 ± 1.3	18135 ± 441	–6.02 ± 0.03	–5.81	0.21

a β-CD, monoSBX, and SBX were injected into the CIT solution in the calorimeter cell in a buffer of 30 mM phosphate, pH 7.4 at 25 °C and the normalized enthalpy curve was fitted with “one set of sites” model. Data are averages of two complete titration series shown separately in Supporting Information Table S3. Because the differences between the repeated experiments were less than the fitting error, the fitting error was indicated as error.

The thermodynamic parameters derived from fittings with the “one set of sites” model are shown in Table 2.

### 3.5 NMR Study of CIT–CD Interactions

NMR is one of the most powerful techniques for studying host–guest interactions at the atomic level, providing deeper insights into the molecular interactions between the tested CDs and the CIT. NMR spectra clearly reveal differences between the real inclusion complexes and other possible interactions. To characterize the complexes, ^1^H NMR, COSY, and HSQC measurements were performed, and the corresponding signal assignments are provided in the Supporting Information. Job’s plot and Scott plot analyses were carried out for the CIT−β-CD and CIT–SBX systems to determine the binding stoichiometry. The results indicate a 1:1 binding ratio for both complexes. Furthermore, Job’s plot diagrams suggest that the interaction between SBX and CIT is stronger than that between β-CD and CIT.

In the case of the β-CD–CIT complex, significant enantiomeric recognition was observed for the aromatic protons and the He proton of CIT. The system was further investigated by spiking the β-CD–CIT complex with *S*-citalopram oxalate, enabling the identification of the *R*- and *S*-enantiomers of CIT by ^1^H NMR spectroscopy. The corresponding spectra are provided in the Supporting Information.

To gain further insight into the structure of these complexes, we performed 1D and 2D ROESY NMR experiments. The 1D ROESY spectra are provided in the Supporting Information, while partial 2D ROESY spectra are presented in Figure 6.

**Figure 6:**
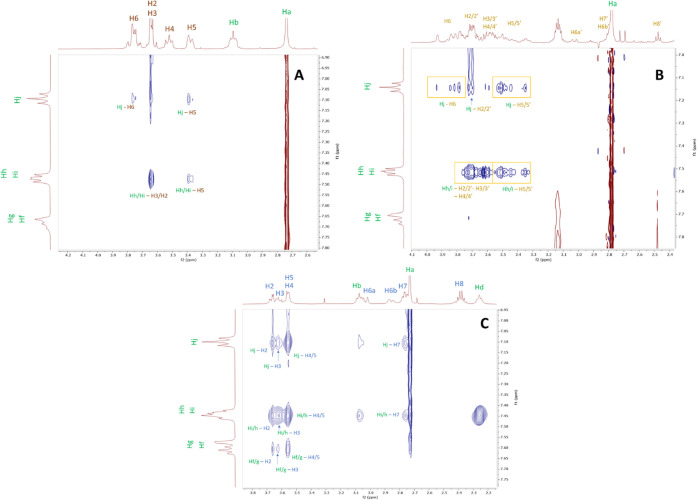
2D ROESY NMR spectrum of (**A**) CIT−β-CD complex, (**B**) CIT–monoSBX complex, and (**C**) CIT–SBX complex. The samples contained 3 mM CD with CIT added in excess to achieve saturation (maximum amount solubilized by the CD) in 30 mM NaH_2_PO_4_ (D_2_O) buffer at pH 7.4 (adjusted with 1 M NaOD). Spectra were recorded at 500 MHz using the residual HOD signal as the chemical shift reference (δ = 4.700 ppm).

ROESY spectra confirmed intermolecular interactions between CIT and all three CDs, as evidenced by cross-peaks between the core protons of the CDs and the aromatic protons of CIT. The β-CD–CIT and monoSBX–CIT complexes exhibited less intense cross-peaks than did the SBX–CIT complex, suggesting greater stability (tighter fit through encapsulation) for the latter. However, less intense cross-peaks are also expected for the asymmetrical monoSBX derivative, where ^1^H resonances are more dispersed. Our findings on the β-CD–CIT complex are consistent with those of Ali et al.,^26,27^ indicating a 1:1 stoichiometry and showing that CIT can adopt multiple orientations within the β-CD cavity, although the most favorable interactions involve the aromatic regions of CIT.

Intermolecular cross-peaks were observed between the H3 and H5 cavity protons of the β-CD and the Hj protons of CIT. Notably, the Hh/i proton of CIT also shows cross-peaks with the H3 and H5 protons of the β-CD. These observations suggest that the fluorinated aromatic ring of CIT is accommodated within the CD cavity most likely entering from the secondary (wider) rim of β-CD. However, due to signal overlap, the exact structure of the complex cannot be determined unambiguously. In the case of the CIT–monoSBX complex, numerous cross-peaks were also observed between the H3/H5 cavity protons, the H2/H4 spherical protons, and the CIT aromatic protons, which confirm the formation of the inclusion complex. However, due to the extensive overlap of the protons in monoSBX, the complex’s 3D structure cannot be unambiguously determined. In the case of the CIT–SBX complex, the aromatic protons of CIT show correlations with the H3/H5 cavity protons of SBX; however, it is important to note that in the 2D ROESY spectrum a further cross-peak was detected with the H7 proton of SBX. This spatial proximity was further confirmed by 1D ROESY experiments. In the case of the CIT–SBX complex, the 1D ROESY spectrum revealed a weak interaction between CIT and the H8 proton, providing experimental evidence that the anionic side chain also participates in complex formation. Due to the extensive overlap of NMR signals, the exact structure of the complex cannot be determined with full certainty. Nevertheless, the formation of an inclusion complex is clearly supported by the experimental data.

## 4 Conclusions

This study aimed to compare different techniques for determining the stability constants of cyclodextrin inclusion complexes. Using β-CD, monoSBX, and SBX as host molecules and citalopram as the guest, we evaluated four methods: phase solubility study, HD, affinity capillary electrophoresis (ACE), and isothermal titration calorimetry (ITC).

The phase solubility study yielded results that deviated from the other three techniques, with monoSBX showing the highest stability constant. The long equilibration time (24 h) in this method allows more interactions to occur, whereas in HD, ACE, and ITC, the interaction time is much shorter and the possibility of the different interactions is lower. The phase solubility method is useful for assessing solubility enhancement, even though it may not always provide accurate stability constants. This observation underlines that the stability and solubility of the formed complex are not directly proportional: a complex with a high stability constant may still display reduced water solubility, and the most stable complex is not always the most soluble one. The HD method proved to be fast and material-efficient, requiring only small amounts of CD. While the absolute stability constants differed from ACE and ITC, the trend in CD stability followed the same order (β-CD < monoSBX < SBX). Additional advantages include high reproducibility and low standard deviation, making it a reliable alternative, especially in laboratories equipped with HPLC but lacking specialized instruments. The ACE and ITC methods yielded highly similar results for β-CD and monoSBX, with some variation for SBX, although still within the same order of magnitude. Both methods confirmed that stability constants increase with the degree of substitution of CDs. Given their accuracy, ACE and ITC appear to be the most reliable techniques for determining CD–guest stability constants. However, their availability is more limited than HPLC-based methods. The ^1^H NMR measurements and ROESY NMR studies supported the findings from HD, ACE, and ITC. The largest chemical shift changes were observed for the CIT–SBX complex, confirming its stronger interaction. Both the Job’s plot and the Scott plot confirmed the stoichiometry of the CIT–CD complexes, indicating a 1:1 binding ratio. In the ROESY spectra, cross-peaks were observed between the H3/H5 cavity protons of the CDs and the aromatic protons of CIT, which confirm the formation of the inclusion complex. However, due to the extensive overlap of the protons of CDs, the 3D structure of the formed complex cannot be unambiguously determined.

In summary, while each method has its advantages and limitations, ACE and ITC provide the most accurate stability constants, HD offers a reliable and resource-efficient alternative, and phase solubility remains useful for solubility enhancement studies, despite its potential inaccuracies in stability determination. The NMR results further validate the ranking of CDs and provide molecular-level insights into complex formation.

## Supplementary Material



## Data Availability

Data will be made available on request.

## Notes

Description of the synthesis of the used CDs and characterization with ^1^H and ^13^C NMR, HPLC-DAD chromatograms for the phase solubility study and the HD method, additional electropherograms for the ACE study and the fitted nonlinear curves, additional thermodynamic parameters and fitted curves for the ITC study, ^1^H, COSY, HSQC, and 1D ROESY NMR characterization of the complexes, Job’s plot analysis, and NMR titration (PDF)
