# Supplementary material for: Complexation of Citalopram with β‑Cyclodextrin, Mono-subetadex, and Subetadex: Phase Solubility, Hummel–Dreyer, Affinity Capillary Electrophoresis, ITC, and NMR Studies
Source: ACS Omega. 2026 Apr 15;11(16):24323–33. doi: 10.1021/acsomega.5c13544 (PMC13130130; doi:10.1021/acsomega.5c13544)
Supplement: Supplementary file 1 [file ao5c13544_si_001.pdf]

Supporting Information
for

Complexation of Citalopram with β-Cyclodextrin, Mono-Subetadex and Subetadex: Phase Solubility, Hummel-Dreyer, Affinity Capillary Electrophoresis, ITC and NMR Studies

Dóra V. Ujj[1], Petr Kasal[2], Ida Fejős[3,4], Szabolcs Béni[5], József Kardos[6], Gábor Benkovics[1], Erika Bálint[1]*, Béla Mátravölgyi[1]*

[1]Department of Organic Chemistry and Technology, Faculty of Chemical Technology and Biotechnology, Budapest University of Technology and Economics, Műegyetem rkp. 3, H-1111 Budapest, Hungary

[2]Institute of Organic Chemistry and Biochemistry, Czech Academy of Sciences, Národni 3, CZ-110 00 Prague 1, Czech Republic

[3]Department of Pharmacognosy, Semmelweis University, Üllői út 26, H-1085 Budapest, Hungary

[4]Center for Pharmacology and Drug Research & Development, Semmelweis University, Üllői út 26, H-1085 Budapest, Hungary

[5]Integrative Health and Environmental Analysis Research Laboratory, Department of Analytical Chemistry, Institute of Chemistry, ELTE Eötvös Loránd University, Pázmány Péter sétány 1/a, H-1117 Budapest, Hungary

[6]ELTE NAP Neuroimmunology Research Group, Department of Biochemistry, ELTE Eötvös Loránd University, Pázmány Péter sétány 1/C, H-1117 Budapest, Hungary

Corresponding Author:

Erika Bálint*: balint.erika@vbk.bme.hu
Béla Mátravölgyi*: bmatravolgyi@edu.bme.hu
Mailing address: Műegyetem rkp. 3, H-1111 Budapest, Hungary
Phone: (+36-1) 463 1111/5886

Table of content

1 Synthesis of CDs ... 3

2 Phase solubility study – supplementary information ... 6

2.1 HPLC method ... 6

2.1.1 Selectivity test ... 6

2.1.2 Linearity test ... 6

2.2 Chiral recognition of CDs ... 7

3 Hummel-Dreyer method – supplementary information ... 8

3.1 Selectivity test ... 8

3.2 Linearity test ... 8

4 Affinity capillary electrophoresis – supplementary information ... 10

5 Isothermal titration calorimetry (ITC) study – supplementary information ... 12

6 NMR study of CIT-CD interactions ... 15

6.1 NMR assignment ... 15

6.2 Assignment of CIT enantiomers ... 20

6.3 Job's plot method ... 22

6.4 NMR titration method ... 25

6.5 1D ROESY NMR study ... 28

7 References ... 31

1 Synthesis of CDs

6^A-*O*-*p*-Toluenesulfonyl-β-CD The compound was prepared according to the previously published procedure.[1] The suspension of β-CD (58.3 g, 51.4 mmol, not dried) and TsCl (14.7 g, 77.0 mmol) in H_2O (1200 mL) was stirred for 2 hours at room temperature. A solution of NaOH (25 g) in H_2O (240 mL) was added. After 10 minutes, unreacted TsCl was separated by filtration, the filtrate was neutralized with 10 M HCl, and the solution was put into a fridge for a night. The resulting precipitate was collected by filtration, washed with ice cold H_2O, and dried at 70 °C using an oil rotary pump. The crude product was purified by repeated recrystallization from H_2O/MeOH 1/1 mixture. The pure product was dried at 70 °C using an oil rotary pump and obtained as a white crystalline solid in an 8% yield (5.6 g). The reaction mixture and purification process was monitored by TLC using n-PrOH/H_2O/EtOAc/conc. NH_3 aq. solution 6/3/1/1 mixture. Spots were detected by an iodine sorbed on silica gel or by dipping TLC plate into 50% V/V H_2SO_4 with subsequent heating.

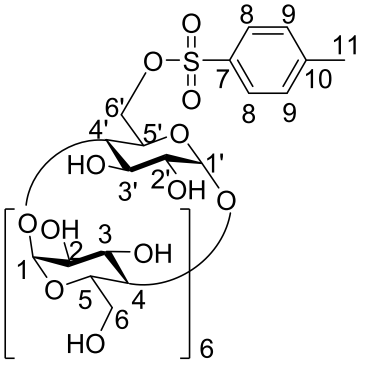

^{1}H NMR (500 MHz, DMSO-d$_6$): δ 7.75 (m, 2H, H-8), 7.43 (m, 2H, H-9), 5.82–5.63 (m, 14H, OH), 4.87–4.75 (m, 7H, H-1, H-1'), 4.52–4.17 (m, 8H, OH, H-6'), 3.72–3.19 (m, 40H, H-2, H-2', H-3, H-3', H-4, H-4', H-5, H-5', H-6, solvent overlay), 2.43 (s, 3H, H-11) ppm.

Per(6-deoxy-6-iodo)-β-CD. Compound per(6-I)-β-CD was prepared according to the previously published procedure.[2] PPh_3 (37.5 g, 0.14 mol) was dissolved in dry DMF (65 mL) in a one-liter, three-necked flask equipped with an argon inlet, a drying tube, and a thermometer. The solution was cooled to approximately 18 °C, and I_2 (38.5 g, 0.15 mol) was added in small portions in a way that the temperature of the solution did not exceed 30 °C. Dried β-CD (10 g, 8.8 mmol) was then added to the obtained brown solution, and the mixture self-heated to 50 °C. The temperature was raised to 70 °C, and the reaction mixture was stirred for 3 hours. The reaction was monitored by TLC using dioxane/n-PrOH/conc. NH_3 aq. solution 10/3/7 mixture. Visualization was done by dipping TLC plate into 50% V/V H_2SO_4 with subsequent heating. The reaction mixture was diluted with MeOH (450 mL). A 25% w/w solution of NaOMe in MeOH was added until the pH was raised to 9, and a precipitate formed. The suspension was stirred overnight. The precipitate was collected by filtration, washed with MeOH (4 × 100 mL), H_2O to neutrality (2 × 100 mL), and again with

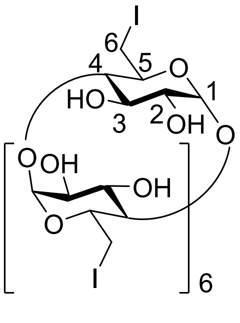

MeOH (100 mL). The product was air-dried and then evacuated at 70 °C for 3 days. The product was obtained as a beige bulk solid in a 49% yield (8.2 g).

^{1}H NMR (500 MHz, DMSO-d$_6$): δ 6.01 (d, 7H, OH-2), 5.91 (br.s. 7H, OH-3), 3.80 (d, 7H, H-6), 3.65 (t, 7H, H-5), 3.59 (t, 7H, H-3), 3.44 (t, 7H, H-6'), 3.37 (dt, 7H, H-2), 3.29 (t, 7H, H-4) ppm.

Mono-subetadex (monoSBX). 3-Mercaptopropionic acid (100 μL, 1.2 mmol) was poured sequentially in DMSO (1.25 mL). Sodium methoxide-methanol solution (30% w/w, 430 μL, 2.3 mmol) was slowly added. Mono-6^A-tosyl-β-CD (0.5 g, 0.4 mmol) in DMSO (10 mL) was stirred for 20 hours at room temperature. Progress of the reaction was checked by TLC with n-PrOH/H_2O/EtOAc/conc. NH_3 aq. solution 6/3/1/1 mixture. Visualization was done by dipping TLC plate into 50% V/V H_2SO_4 with subsequent heating. Acetone (200 mL) was added into reaction and the resulting precipitate was stirred for 1h. Solid crude was collected by filtration and washed with acetone (3 × 100 mL), and was dried at 60 °C using an oil rotary pump. Crude (0.63 g) was dissolved in H_2O (12 mL), silica gel (3 g) was added, and mixture was evaporated on a rotary evaporator at 50 °C. Adsorbed crude was purified by CC (16 g of silica gel) using n-PrOH/H_2O/ conc. NH_3 aq. solution 15/7/3. All fractions containing the product were combined and evaporated on a rotary evaporator at 50 °C. Product (0.22 g) was dissolved in H_2O (3 mL) and freeze-dried. The product was obtained as a white solid (0.17 g) in 36% yield.

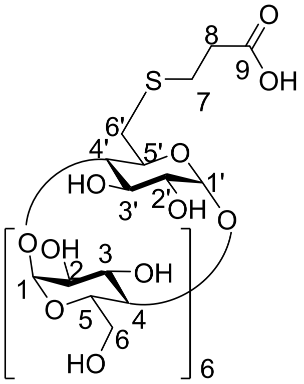

^{1}H NMR (500 MHz, DMSO-*d*$_6$) δ 5.72 (bs, 14H, OH), 4.82 (m, 7H, H-1, H-1'), 3.86 – 3.50 (m, 26H, H-3, H-3', H-5, H-5', H-6), 3.46 – 3.20 (m, 14H, H-2, H-2', H-4, H-4'), 3.02 (d, J = 13.6 Hz, 1H, H-6'), 2.66 (m, 3H, H-6', H-7), 2.34 (t, J = 7.6 Hz, 2H, H-8) ppm.

^{13}C NMR (126 MHz, DMSO-*d*$_6$) δ 174.19 (C-9), 104.22 – 100.50 (C-1, C-1'), 85.68 – 80.36 (C-4, C-4'), 74.32 – 71.09 (C-2, C-2', C-3, C-3', C-5, C-5'), 65.00 – 58.51 (C-6), 36.30 (C-8), 33.59 (C-6'), 28.25 (C-7) ppm.

Subetadex. 3-Mercaptopropionic acid (7.8 mL, 89.3 mmol) was dissolved in DMSO (20 mL) and solution was cooled down to 15 °C. Sodium methoxide-methanol solution (30% w/w, 22 mL, 0.119 mol) was slowly added. Per(6-deoxy-6-iodo)-β-CD (8.1 g, 4.3 mmol) was dissolved in DMSO (140 mL), added into 3-mercaptopropionic acid solution, and reaction mixture was stired at RT for 1 hour. Progress of the reaction was checked by TLC with dioxane/n-PrOH/ conc. NH_3 aq. solution 10/3/7 mixture. Visualization was done by dipping TLC plate into 50% V/V H_2SO_4 with subsequent heating. MeOH (160 mL) was added into reaction and the resulting precipitate was stirred for 1 hour. The reaction mixture was filtered and the solid was washed with methanol until a white solid was obtained. The solid was placed into a drying box and dried until constant weight. The product was obtained as a white solid (3.2 g) in 39.5% yield.

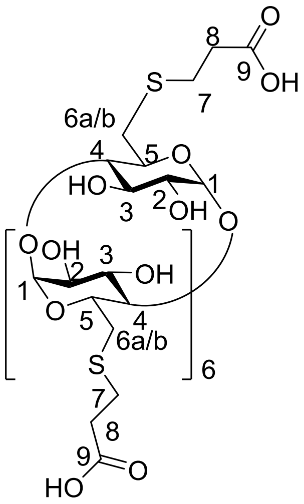

^{1}**H NMR** (500 MHz, D_2O) δ 5.20 (s, 7H, H-1), 4.04 (t, J = 8.4 Hz, 7H, H-5), 3.98 (t, J = 9.4 Hz, 7H, H-3), 3.69 – 3.61 (m, 14H, H-2, H-4), 3.18 (d, J = 14.2 Hz, 7H, H-6a), 3.00 (dd, J = 13.9 Hz, 7H, H-6b), 2.88 (t, J = 7.4 Hz, 14H, H-7), 2.54 (t, J = 7.3 Hz, 14H, H-8) ppm.

^{13}**C NMR** (126 MHz, D_2O) δ 176.01 (C-9), 100.23 (C-1), 84.26 (C-4), 72.62-70.82 (C-2, C-3, C-5), 37.35 (C-8), 33.12 (C-6), 29.11 (C-7) ppm.

2 Phase solubility study – supplementary information

2.1 HPLC method

2.1.1 Selectivity test

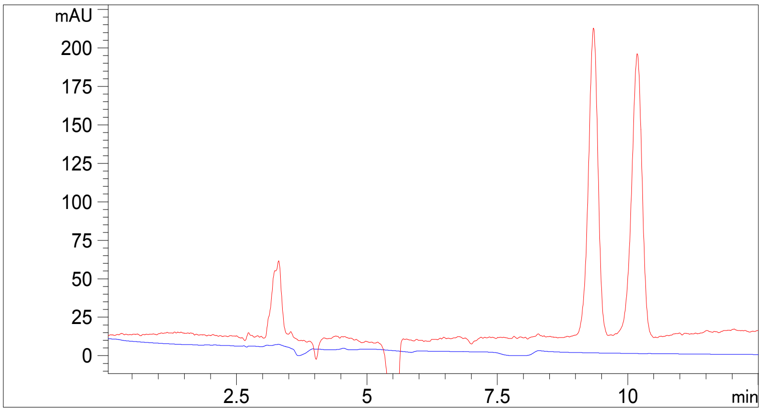

Figure S1. Separation of the two CIT enantiomers with the used HPLC method and selectivity test for the HPLC quantification method (Lux Cellulose-1 (250 mm · 4.6 mm, 5 µm), isocratic elution: ACN:PW = 60:40 + 0.1% DEA, flow rate: 0.8 mL/min, injection volume: 10 µL, column temperature: 25°C, detection at 222 nm). Blank (ACN : PW = 60 : 40 + 0.1% DEA) – blue, CIT sample (*LIN 6*) – red.

2.1.2 Linearity test

Citalopram (CIT) stock solution for linearity test: app. 15 mg of CIT was measured into a 10 mL graduated glass flask and made up to the mark with HPLC grade ACN and mix thoroughly with a vortex mixer and labelled as *CIT-stock solution* (concentration: app. 1.5 mg/mL). The Linearity samples 1-8 (*LIN 1-8*) was prepared from *CIT-stock solution* with dilution in the range of 0.03 and 1.3 mg/mL, the diluent solvent was ACN : PW = 60 : 40 + 0.1% DEA. The samples were mixed thoroughly with a vortex mixer.

Table-S1. Raw data of linearity samples.

Sample	c / [mg/ml]	Area
LIN 1	0.03	209.3
LIN 2	0.07	410.2
LIN 3	0.08	562.1
LIN 4	0.16	1054.6
LIN 5	0.33	2099.3
LIN 6	0.65	4174.9
LIN 7	1.09	7105.0
LIN 8	1.30	8406.9

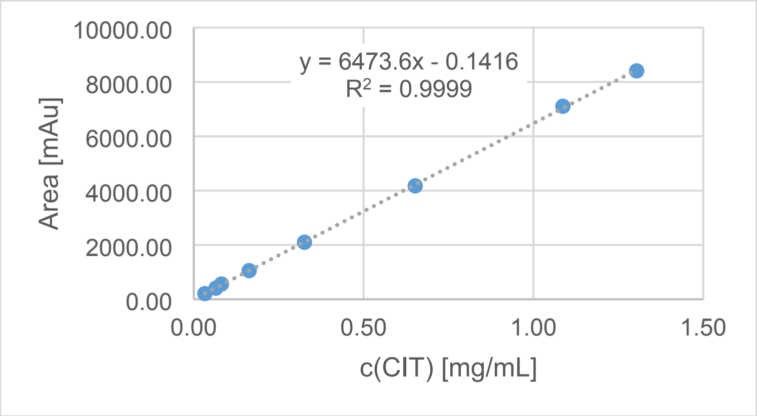

Figure-S2. Linearity curves in the tested (0.03 – 1.30 mg/mL) concentration rage.

Based on the results the calibration is linear in the tested concentration range (0.03 – 1.30 mg/mL).

2.2 Chiral recognition of CDs

The HPLC measurements were carried out on a chiral column to investigate the ratio of the citalopram enantiomers. The peak ratio of the two enantiomers of CIT is 1:1, therefore no chiral recognition occurred in the course of phase solubility tests.

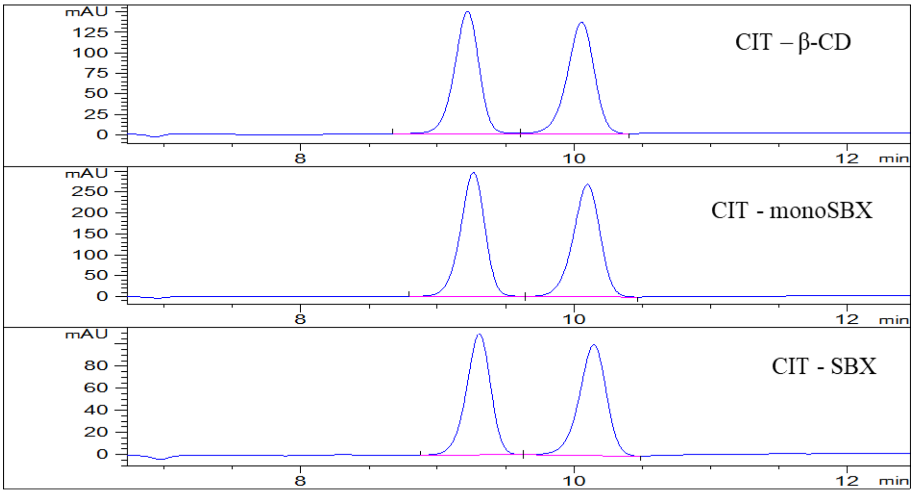

Figure S3. Ratio of the enantiomers: CIT-β-CD: first peak: 1888.9 mAu, second peak: 1888.0 mAu; CIT-monoSBX: first peak: 3748.3 mAu, second peak: 3741.6 mAu, CIT-SBX: first peak: 1376.5 mAu, second peak: 1411.7 mAu.

3 Hummel-Dreyer method – supplementary information

3.1 Selectivity test

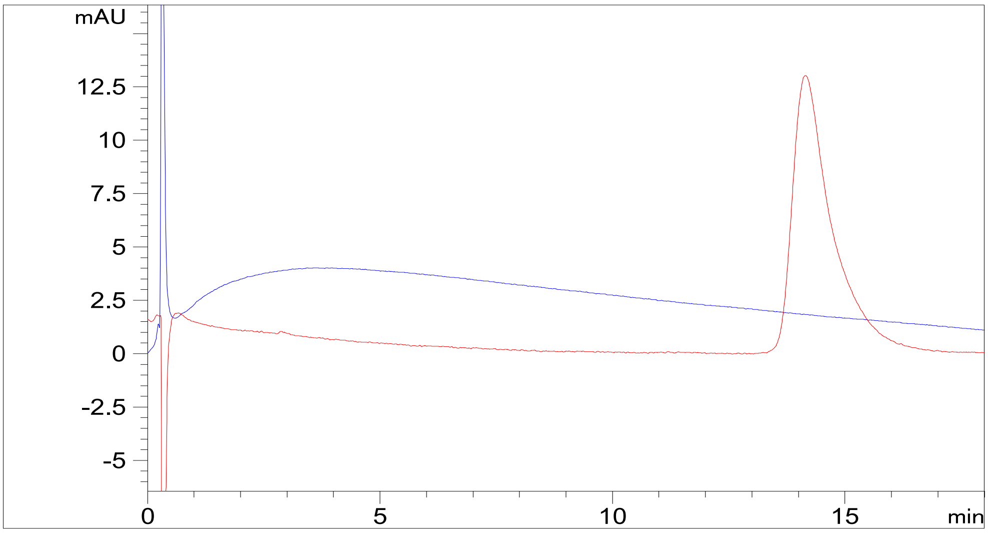

Figure S4. Selectivity test for the utilized HPLC method (YMC-Pack C4 (100 X 2.1 mm, 5 µm, 30 nm), isocratic elution: ACN:30 mM phosphate buffer (pH 7.4) = 10:90 + 0.03 mM CIT, flow: 1.2 mL/min, column temperature: 45 °C, injection: 10 µL, detection at 222 nm). Blank (ACN:30 mM phosphate buffer (pH 7.4) = 10:90) – blue, CIT sample (*LIN 2*) – red.

3.2 Linearity test

Citalopram (CIT) stock solution for linearity test: app. 5 mg of CIT was measured into a 10 mL graduated glass flask and 1.0 mL ACN was added and made up to the mark with 30 mM phosphate buffer (pH 7.4), and mix thoroughly with a vortex mixer and labelled as *CIT-stock solution* (concentration: app. 0.5 mg/mL). The Linearity samples 1-6 (*LIN 1-6*) was prepared from *CIT-stock solution* with dilution in the range of 0.03 and 0.63 mM, the diluent was ACN:30 mM phosphate buffer (pH 7.4) = 10:90. The samples were mixed thoroughly with a vortex mixer. For this test the concentration of the samples was lower due to the lower solubility of CIT in ACN:30 mM phosphate buffer (pH 7.4) = 10:90 solvent.

Table-S2. Raw data of linearity samples.

Sample	c / [mM]	Area
LIN 1	0.03	28.8
LIN 2	0.06	84.4
LIN 3	0.13	161.0
LIN 4	0.21	296.5
LIN 5	0.31	440.6
LIN 6	0.63	891.1

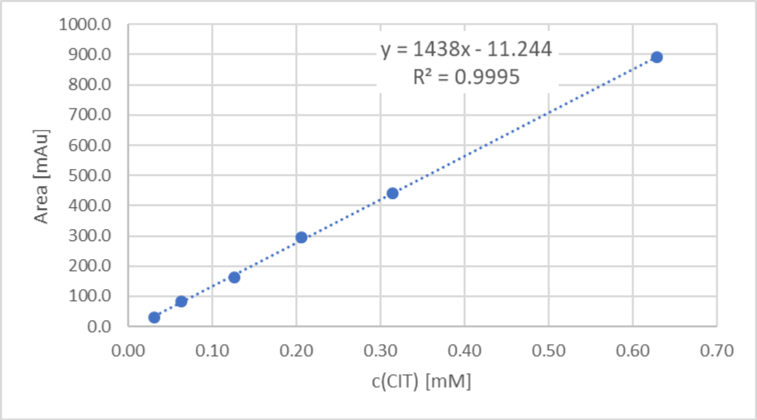

Figure S5. Linearity curves in the tested (0.03 – 0.63 mM) concentration range.

4 Affinity capillary electrophoresis – supplementary information

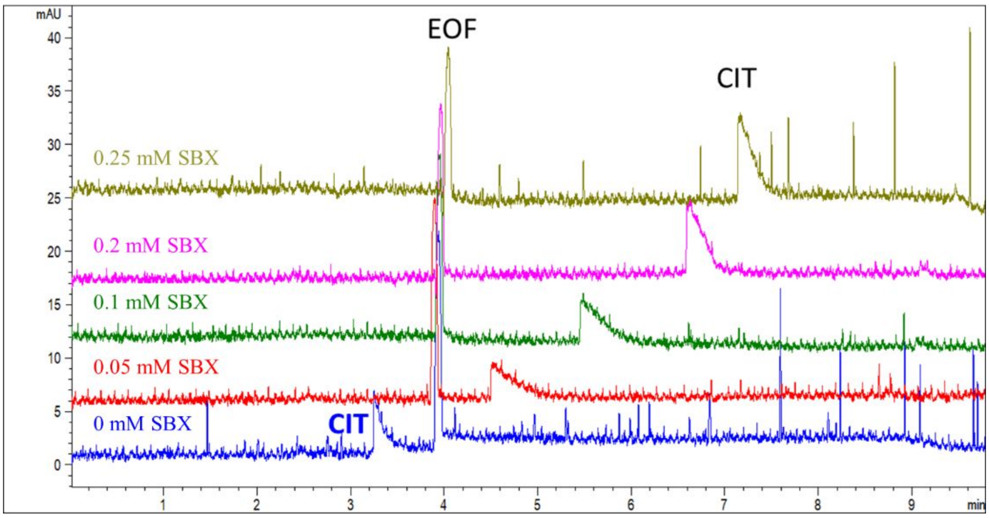

Figure S6. Overlaid electropherograms of CIT applying low concentration of SBX (0.05 – 0.25 mM) in 30 mM phosphate buffer pH 7.4 (Conditions: 50 µm untreated fused silica capillary, 48.5 cm/40 cm; 25°C; + 15 kV; 200 nm).

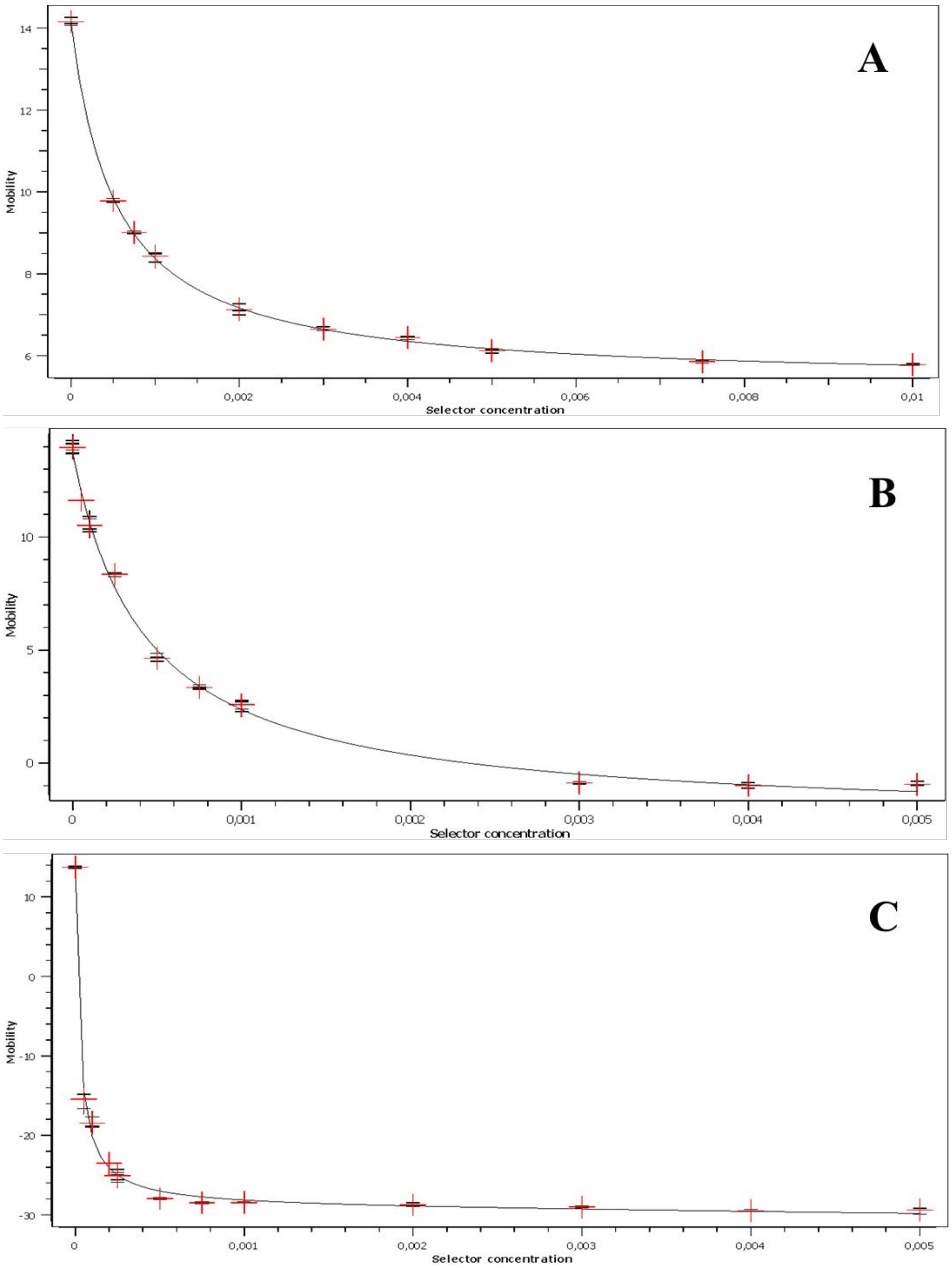

Figure S7. The fitted nonlinear curves for CIT-β-CD (A), CIT-monoSBX (B), CIT-SBX (C).

5 Isothermal titration calorimetry (ITC) study – supplementary information

Table S3. Thermodynamic parameters of CIT-CD complex formation by ITC.

Samples[a]	K_D (μM)	K_A (M^{-1})	ΔH (kcal/mol)	ΔG (kcal/mol)	$-T\Delta S$ (kcal/mol)
10 mM β-CD → 0.5 mM CIT	531±26	1883±99	-4.38±0.17	-4.47	-0.09
10 mM β-CD → 1 mM CIT	559±11	1789±35	-4.34±0.04	-4.44	-0.10
5.6 mM monoSBX → 0.5 mM CIT	424±42	2258±257	-4.87±0.34	-4.60	0.27
5.6 mM monoSBX → 1 mM CIT	449±18	2227±91	-4.94±0.07	-4.57	0.37
20 mM SBX → 1mM CIT	54.5±1.2	18349±410	-5.99±0.02	-5.82	0.17
20 mM SBX → 1mM CIT	55.8±1.3	17921±441	-6.05±0.03	-5.80	0.25

[a]Individual ITC measurements of CIT-CD complex formation. CDs were injected to the CIT solutions with the given concentrations. Thermodynamic parameters were determined by fitting to the normalized enthalpy changes applying a "one type of binding sites" model. Stoichiometry provided by the fitting was consistent with 1:1 complex formation.

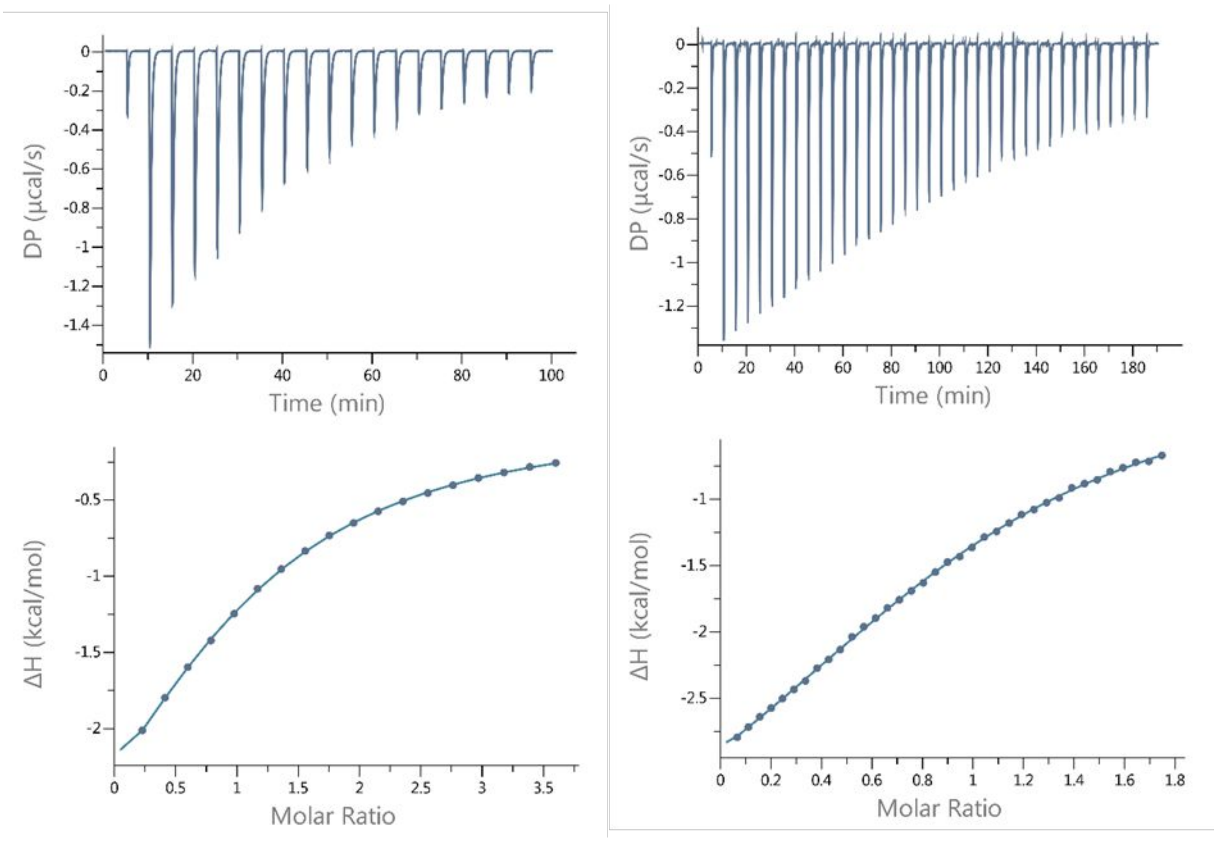

Figure S8. CIT-β-CD complexation studied by ITC. (Left) 10 mM β-CD was injected to 0.5 mM CIT, (right) 10 mM β-CD was injected to 1 mM CIT in 30 mM phosphate buffer, pH 7.4, at 25 °C. (Top) Titration curves, (bottom) normalized enthalpy changes after subtraction of the β-CD→buffer control measurement and fitted curves as a function of the CD:CIT molar ratio.

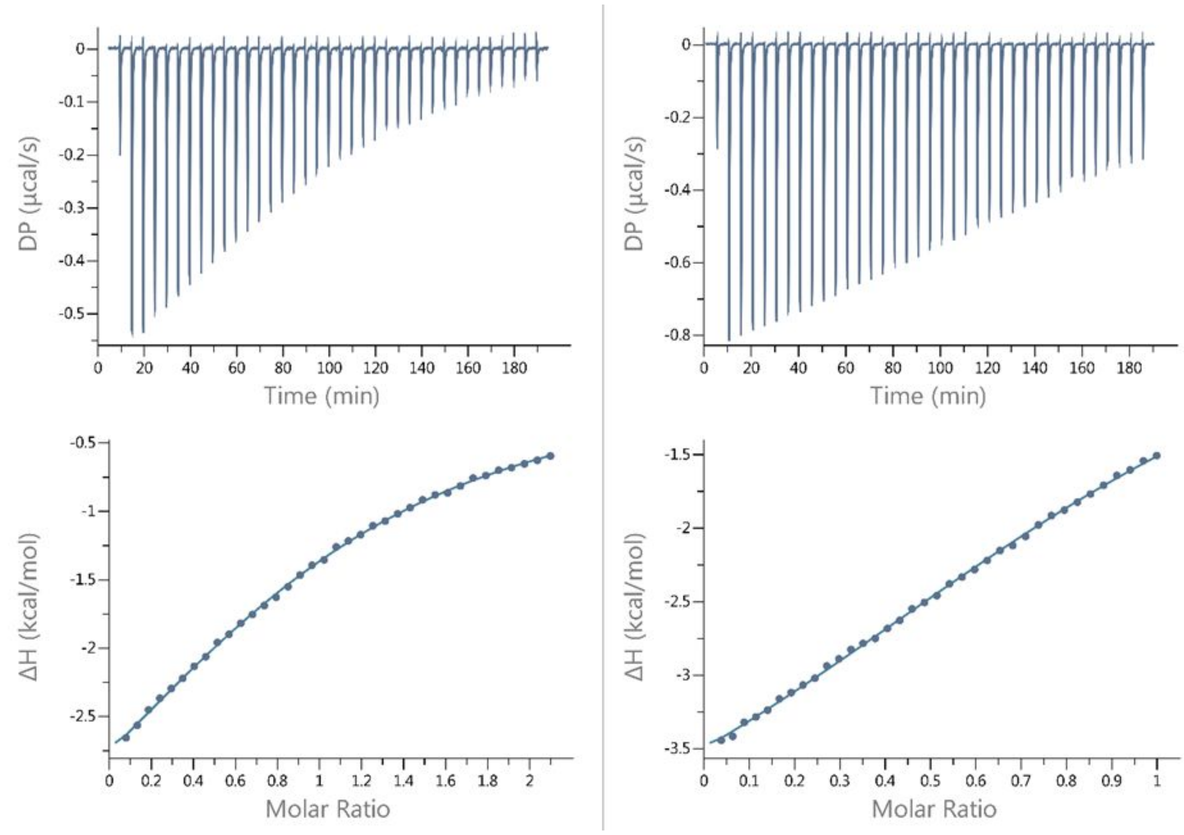

Figure S9. CIT-monoSBX complexation studied by ITC. (Left) 5.6 mM monoSBX was injected to 0.5 mM CIT, (right) 5.6 mM monoSBX was injected to 1 mM CIT in 30 mM phosphate buffer, pH 7.4, at 25 °C. (Top) Titration curves, (bottom) normalized enthalpy changes after subtraction of the monoSBX→buffer control measurement and fitted curves as a function of the CD:CIT molar ratio.

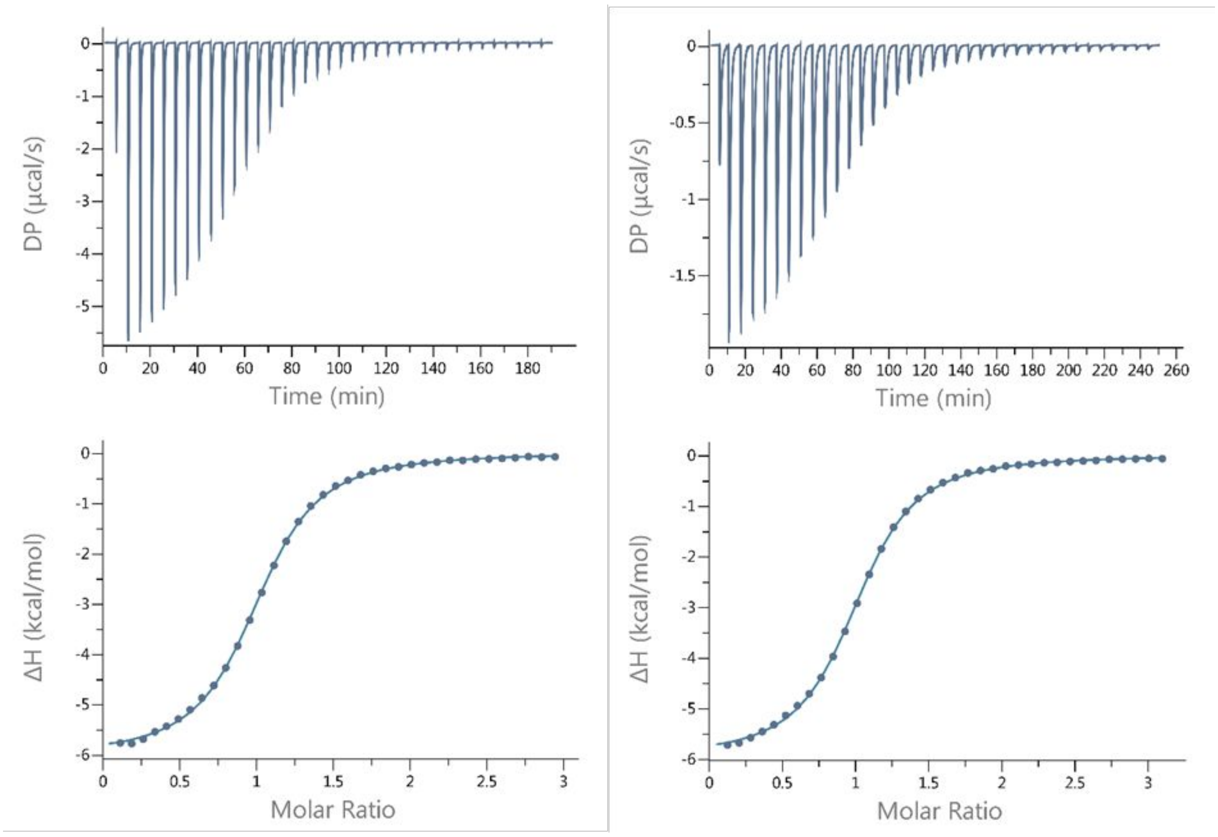

Figure S10. CIT-SBX complexation studied by ITC. 20 mM SBX was injected to 1 mM CIT in 30 mM phosphate buffer, pH 7.4, at 25 °C, repeated experiments. (Top) Titration curves, (bottom) normalized enthalpy changes after subtraction of the SBX→buffer control measurement and fitted curves as a function of the CD:CIT molar ratio.

6 NMR study of CIT-CD interactions

6.1 ^{1}H NMR assignment

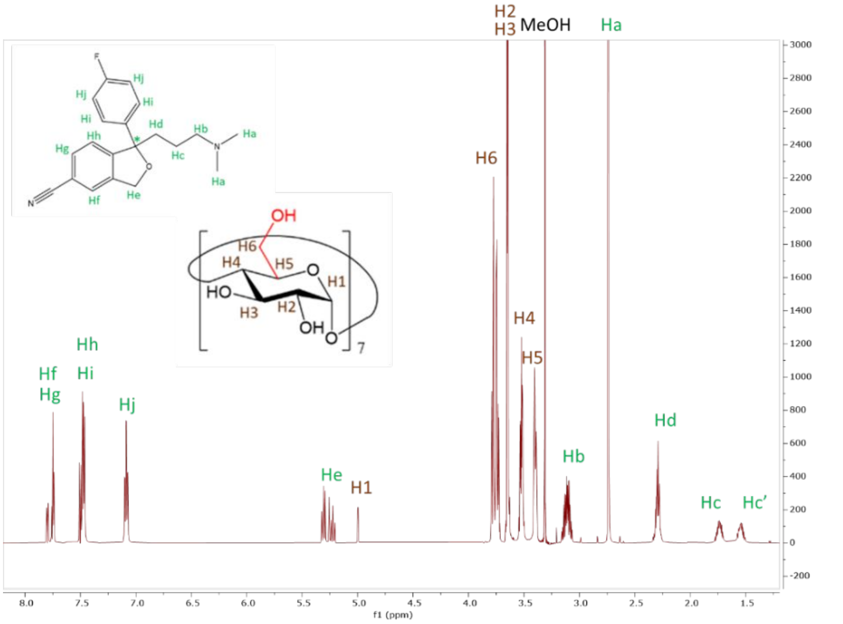

Figure S11. ^{1}H NMR spectrum of the CIT–β-CD complex using excitation sculpting pulse sequence for HDO signal suppression. The sample contained 3 mM β-CD with CIT added in excess to achieve saturation (maximum amount solubilized by β-CD) in 30 mM NaH_2PO_4 (D_2O) buffer at pH 7.4 (adjusted with 1 M NaOD). Spectrum was recorded at 700 MHz using MeOH as an internal reference (δ = 3.310 ppm).

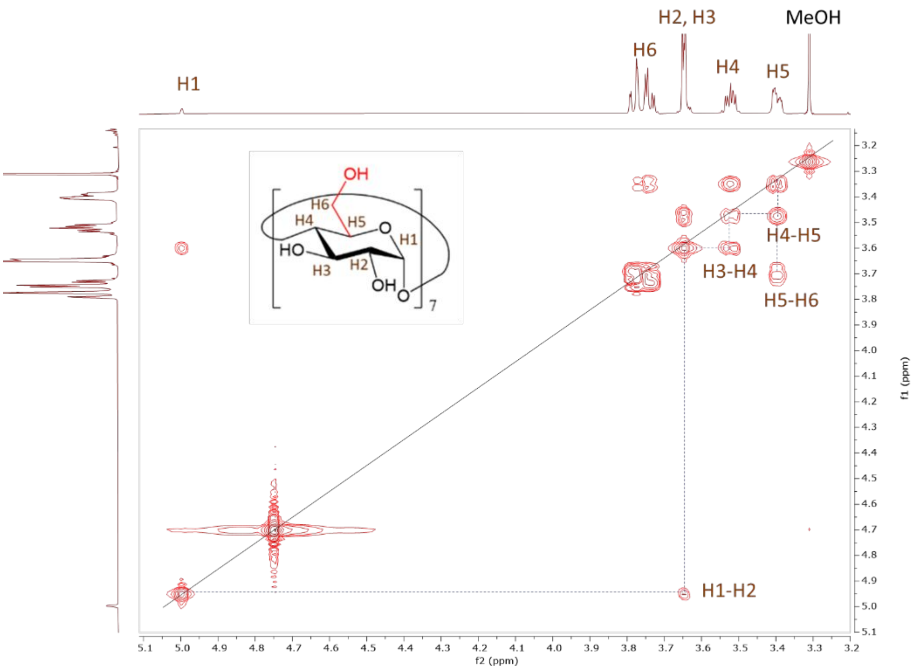

Figure S12. COSY NMR spectrum of the CIT–β-CD complex. The sample contained 3 mM β-CD with CIT added in excess to achieve saturation (maximum amount solubilized by β-CD) in 30 mM NaH_2PO_4 (D_2O) buffer at pH 7.4 (adjusted with 1 M NaOD). Proton assignments of β-CD are indicated. Spectrum was recorded at 700 MHz using MeOH as an internal reference (δ = 3.310 ppm).

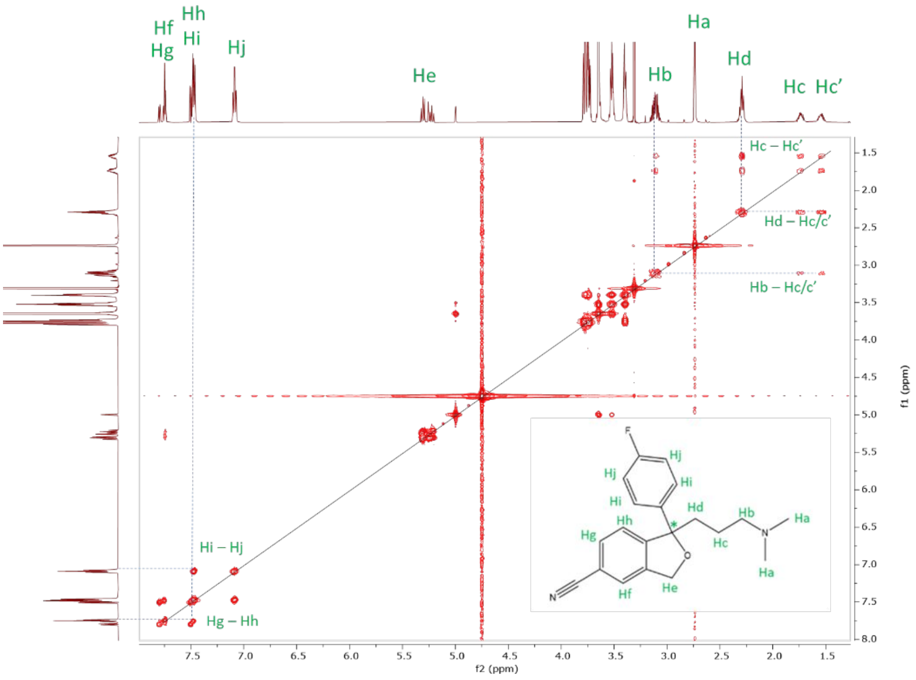

Figure S13. COSY NMR spectrum of the CIT–β-CD complex. The sample contained 3 mM β-CD with CIT added in excess to achieve saturation (maximum amount solubilized by β-CD) in 30 mM NaH_2PO_4 (D_2O) buffer at pH 7.4 (adjusted with 1 M NaOD). Proton assignments of CIT are indicated. Spectrum was recorded at 700 MHz using MeOH as an internal reference (δ = 3.310 ppm).

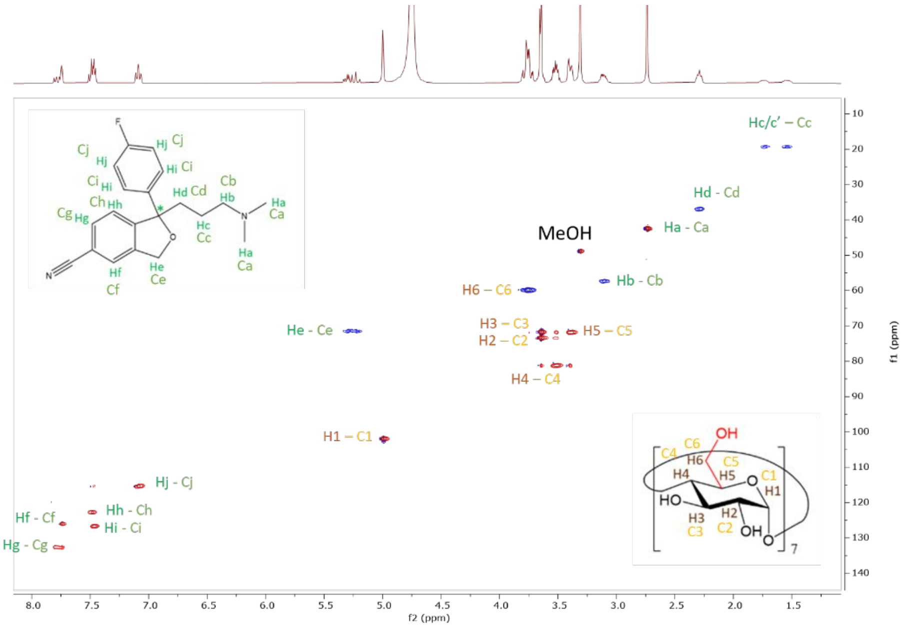

Figure S14. HSQC NMR spectrum of the CIT–β-CD complex. The sample contained 3 mM β-CD with CIT added in excess to achieve saturation (maximum amount solubilized by β-CD) in 30 mM NaH_2PO_4 (D_2O) buffer at pH 7.4 (adjusted with 1 M NaOD). Spectrum was recorded at 400 MHz using MeOH as an internal reference (δ = 3.310 ppm).

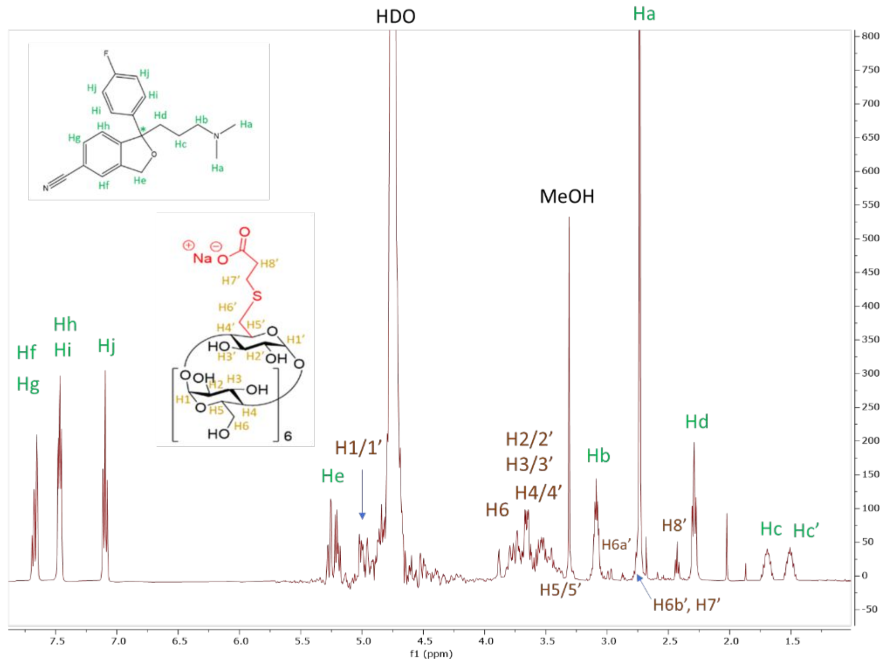

Figure S15. ^{1}H NMR spectrum of the CIT–monoSBX complex. The sample contained 3 mM monoSBX with CIT added in excess to achieve saturation (maximum amount solubilized by monoSBX) in 30 mM NaH_2PO_4 (D_2O) buffer at pH 7.4 (adjusted with 1 M NaOD). Spectrum was recorded at 500 MHz using MeOH as an internal reference (δ = 3.310 ppm).

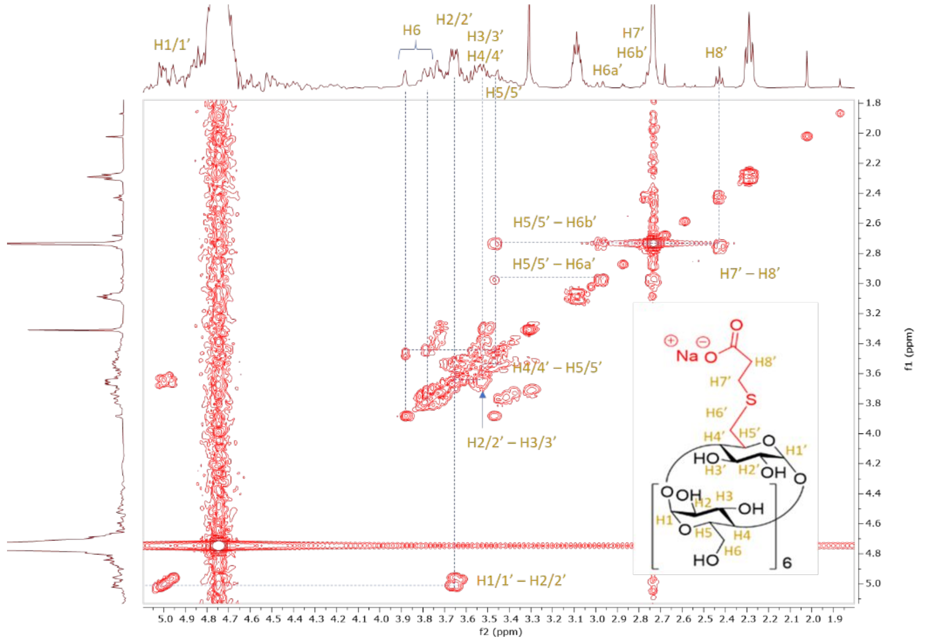

Figure S16. COSY NMR spectrum of the CIT– monoSBX complex. The sample contained 3 mM monoSBX with CIT added in excess to achieve saturation (maximum amount solubilized by monoSBX) in 30 mM NaH_2PO_4 (D_2O) buffer at pH 7.4 (adjusted with 1 M NaOD). Proton assignments of monoSBX are indicated. Spectrum was recorded at 500 MHz using MeOH as an internal reference (δ = 3.310 ppm).

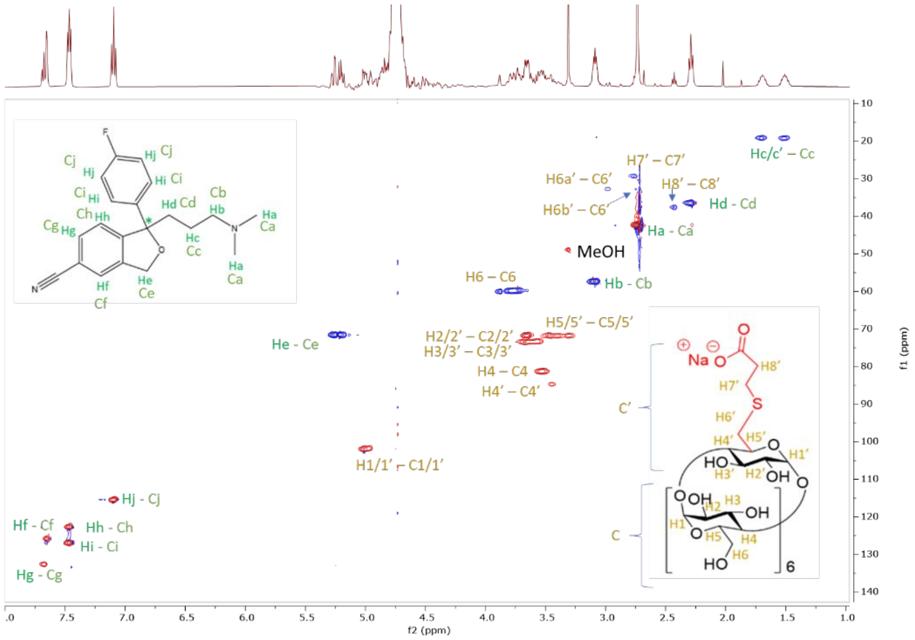

Figure S17. HSQC NMR spectrum of the CIT– monoSBX complex. The sample contained 3 mM monoSBX with CIT added in excess to achieve saturation (maximum amount solubilized by monoSBX) in 30 mM NaH_2PO_4 (D_2O) buffer at pH 7.4 (adjusted with 1 M NaOD). Spectrum was recorded at 500 MHz using MeOH as an internal reference (δ = 3.310 ppm).

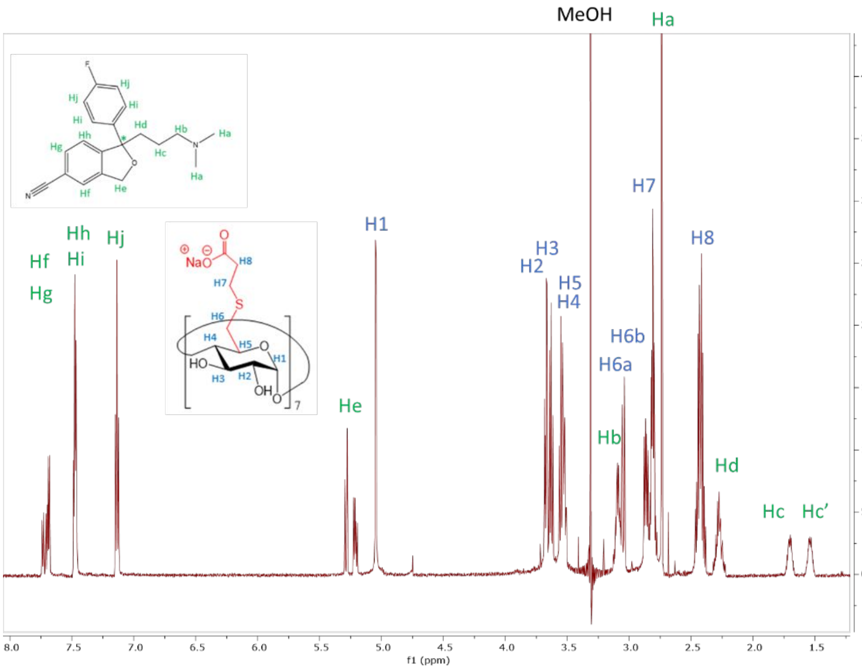

Figure S18. 1H NMR spectrum of the CIT– SBX complex using excitation sculpting pulse sequence for HDO signal suppression. The sample contained 3 mM SBX with CIT added in excess to achieve saturation (maximum amount solubilized by SBX) in 30 mM NaH_2PO_4 (D_2O) buffer at pH 7.4 (adjusted with 1 M NaOD). Spectrum was recorded at 700 MHz using MeOH as an internal reference (δ = 3.310 ppm).

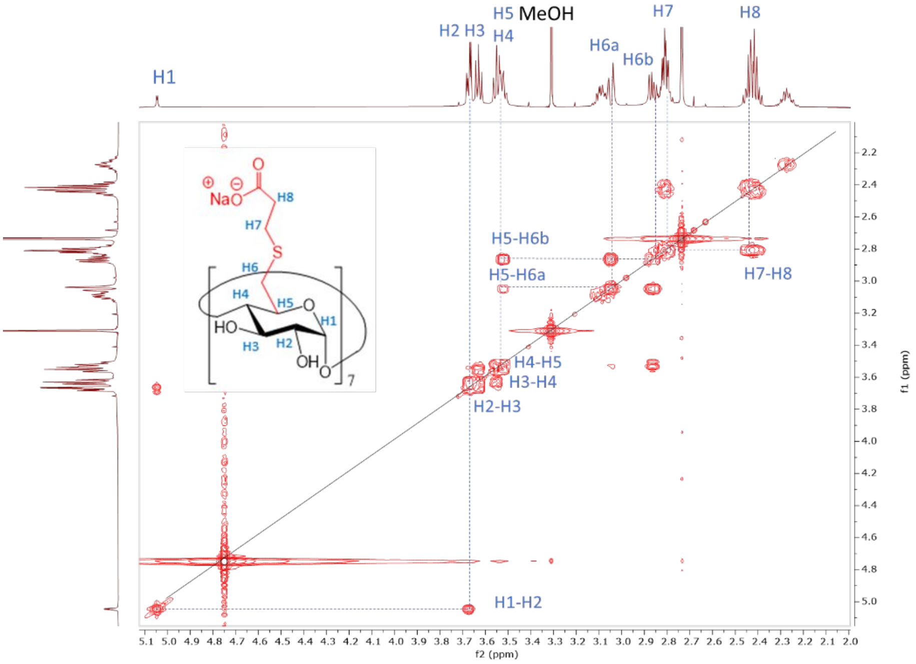

Figure S19. COSY NMR spectrum of the CIT–SBX complex. The sample contained 3 mM SBX with CIT added in excess to achieve saturation (maximum amount solubilized by SBX) in 30 mM NaH_2PO_4 (D_2O) buffer at pH 7.4 (adjusted with 1 M NaOD). Proton assignments of SBX are indicated. Spectrum was recorded at 700 MHz using MeOH as an internal reference (δ = 3.310 ppm).

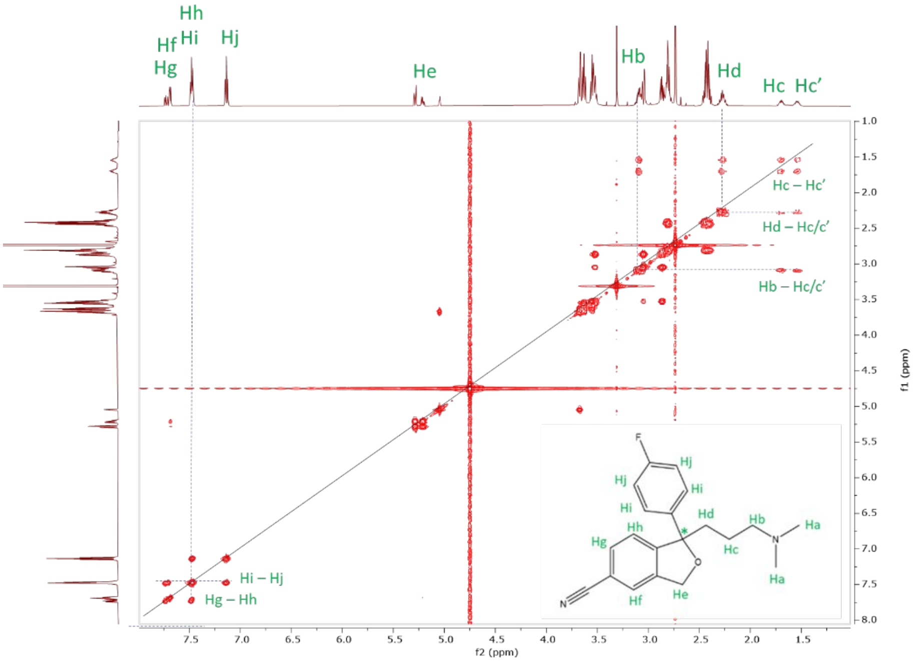

Figure S20. COSY NMR spectrum of the CIT– SBX complex. The sample contained 3 mM SBX with CIT added in excess to achieve saturation (maximum amount solubilized by SBX) in 30 mM NaH_2PO_4 (D_2O) buffer at pH 7.4 (adjusted with 1 M NaOD). Proton assignments of CIT are indicated. Spectrum was recorded at 700 MHz using MeOH as an internal reference (δ = 3.310 ppm).

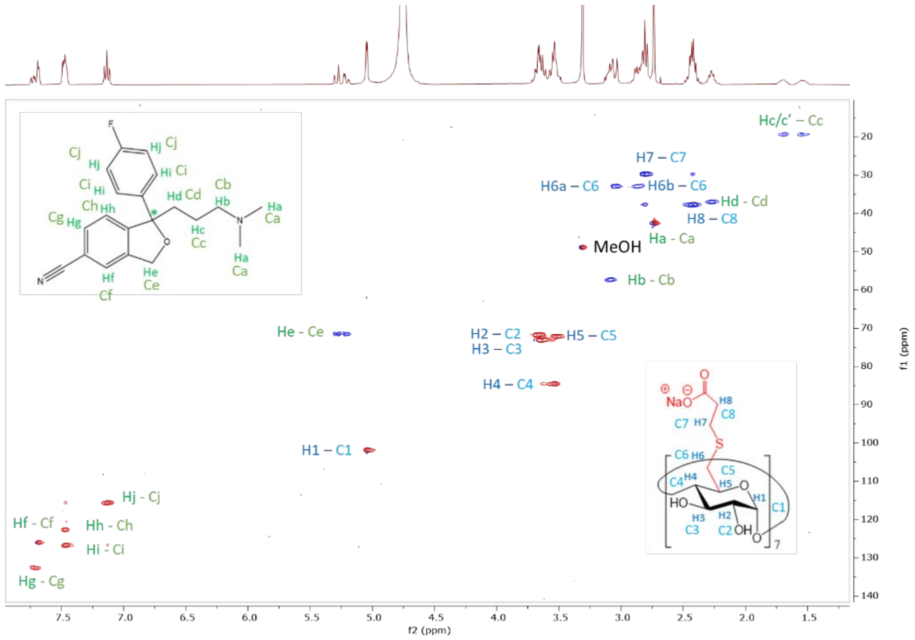

Figure S21. HSQC NMR spectrum of the CIT– SBX complex. The sample contained 3 mM SBX with CIT added in excess to achieve saturation (maximum amount solubilized by SBX) in 30 mM NaH_2PO_4 (D_2O) buffer at pH 7.4 (adjusted with 1 M NaOD). Spectrum was recorded at 400 MHz using MeOH as an internal reference (δ = 3.310 ppm).

6.2 ^{1}H NMR assignment of citalopram enantiomers in CD complexes

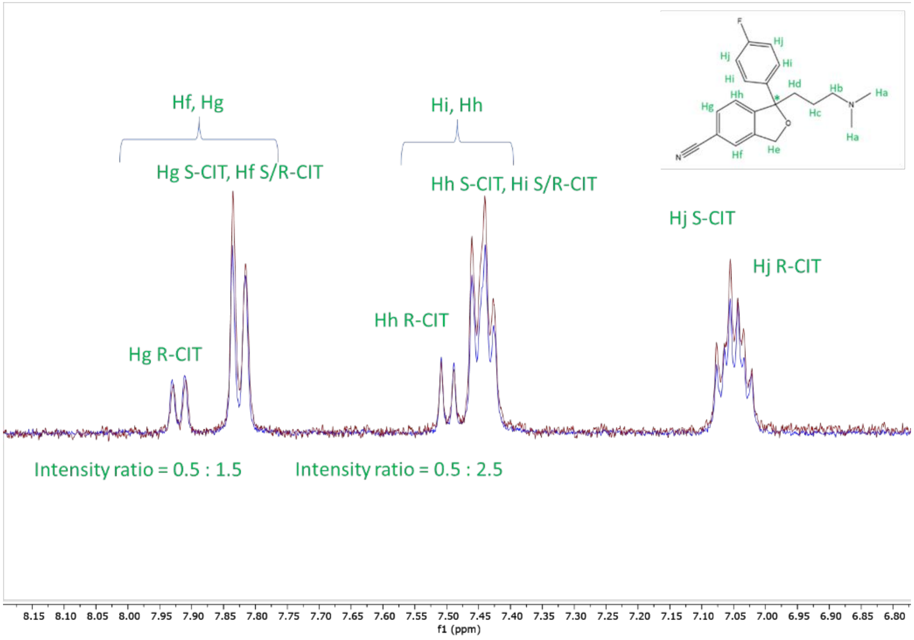

Figure S22. Overlaid ^{1}H NMR spectra (aromatic region) of the CIT–β-CD complex (1:15 molar ratio, blue) and the same sample spiked with S-CIT oxalate (red). Samples were prepared in 30 mM NaH_2PO_4 buffer (10% D_2O) at pH 7.4 (adjusted with 1 M NaOH). Enantiomeric assignment of the Hh and Hi protons was achieved based on intensity ratios. Spectra were recorded at 400 MHz using MeOH as an internal reference (δ = 3.310 ppm).

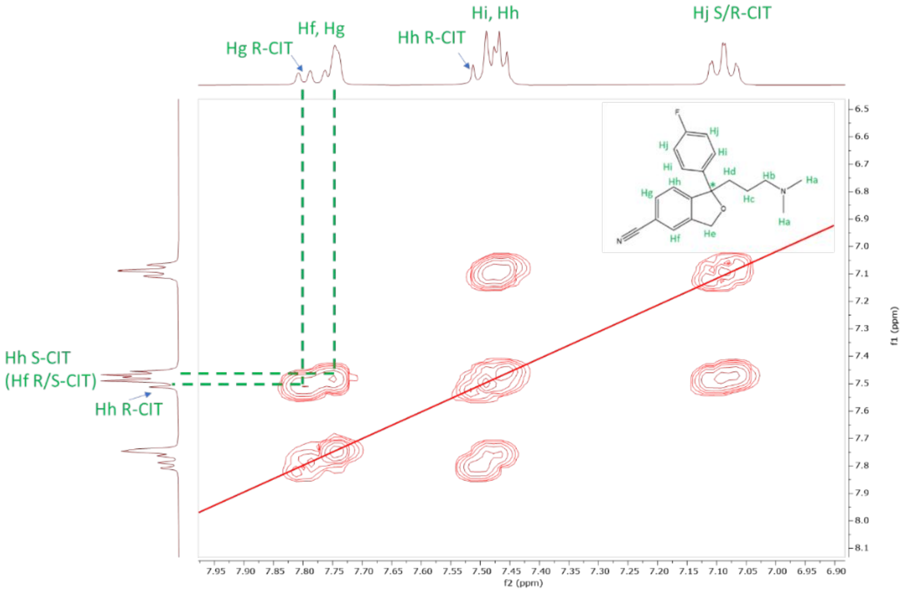

Figure S23. Enantiomeric assignment based on COSY spectrum. Sample: 3mM β-CD + CIT added in excess to reach saturation (maximum amount solubilized by β-CD) in 30 mM NaH_2PO_4 (D_2O), with the pH adjusted to 7.4 using 1 M NaOD. The corresponding Hg Hh and Hf protons were unequivocally identified based on the indicated COSY cross-peaks. Spectrum was recorded at 400 MHz using MeOH as an internal reference (δ = 3.310 ppm).

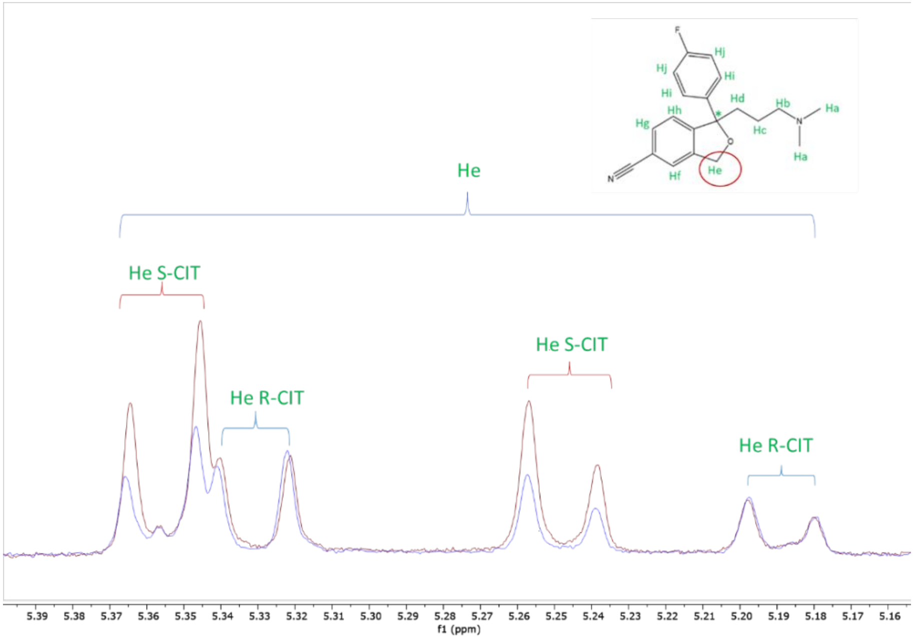

Figure S24. Overlaid ^{1}H NMR spectra of the strongly coupled He protons of CIT in the presence of β-CD (1:10 CIT- β-CD molar ratio, blue) and the same sample spiked with S-CIT oxalate (red) were prepared in 30 mM NaH_2PO_4 buffer (10% D_2O) at pH 7.4 (adjusted with 1 M NaOH). Spectra were recorded at 700 MHz using MeOH as an internal reference (δ = 3.310 ppm).

6.3 Job's plot method

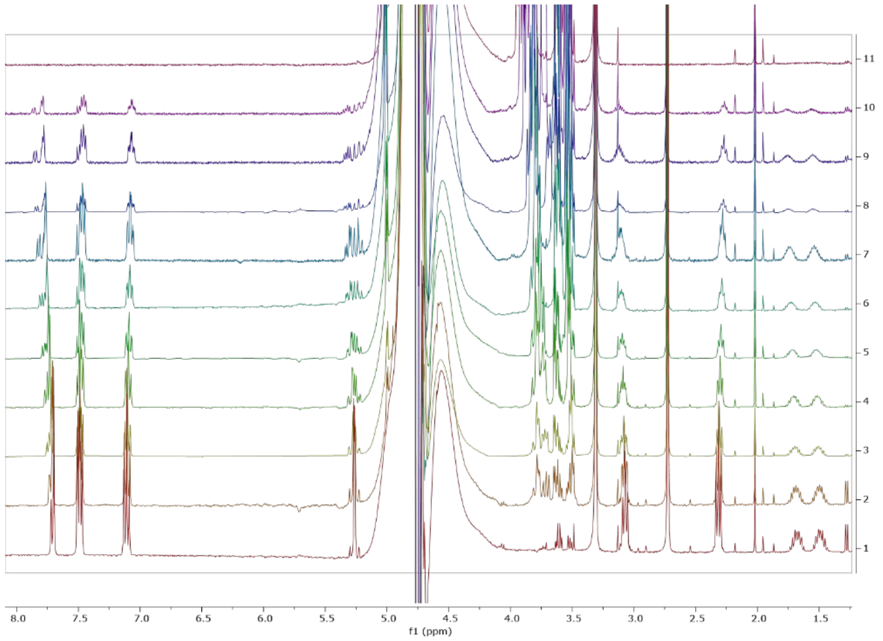

Figure S25. ^{1}H NMR spectra of CIT–β-CD samples recorded for Job's plot analysis (full spectra). The samples were prepared in 30 mM NaH_2PO_4 (10% D_2O), with the pH adjusted to 7.4 using 1 M NaOH. Spectra were recorded at 400 MHz using MeOH as an internal reference (δ = 3.310 ppm).

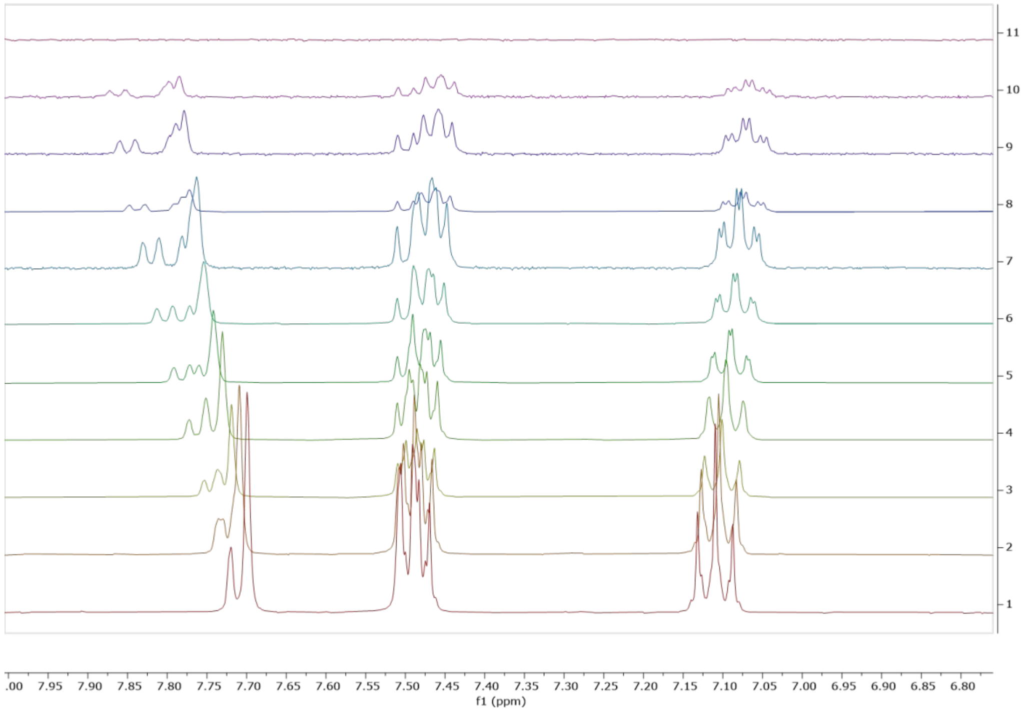

Figure S26. ^{1}H NMR spectra of CIT–β-CD samples recorded for Job's plot analysis (aromatic region). The samples were prepared in 30 mM NaH_2PO_4 (10% D_2O), with the pH adjusted to 7.4 using 1 M NaOH. Spectra were recorded at 400 MHz using MeOH as an internal reference (δ = 3.310 ppm).

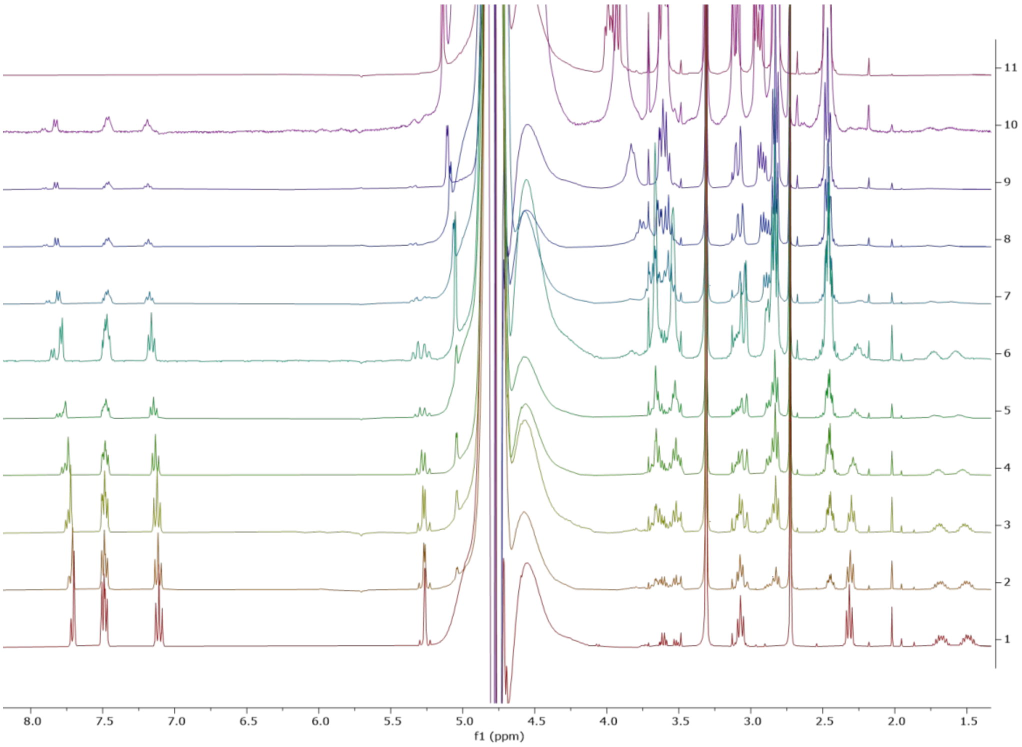

Figure S27. ^{1}H NMR spectra of CIT–SBX samples recorded for Job's plot analysis (full spectra). The samples were prepared in 30 mM NaH_2PO_4 (10% D_2O), with the pH adjusted to 7.4 using 1 M NaOH. Spectra were recorded at 400 MHz using MeOH as an internal reference (δ = 3.310 ppm).

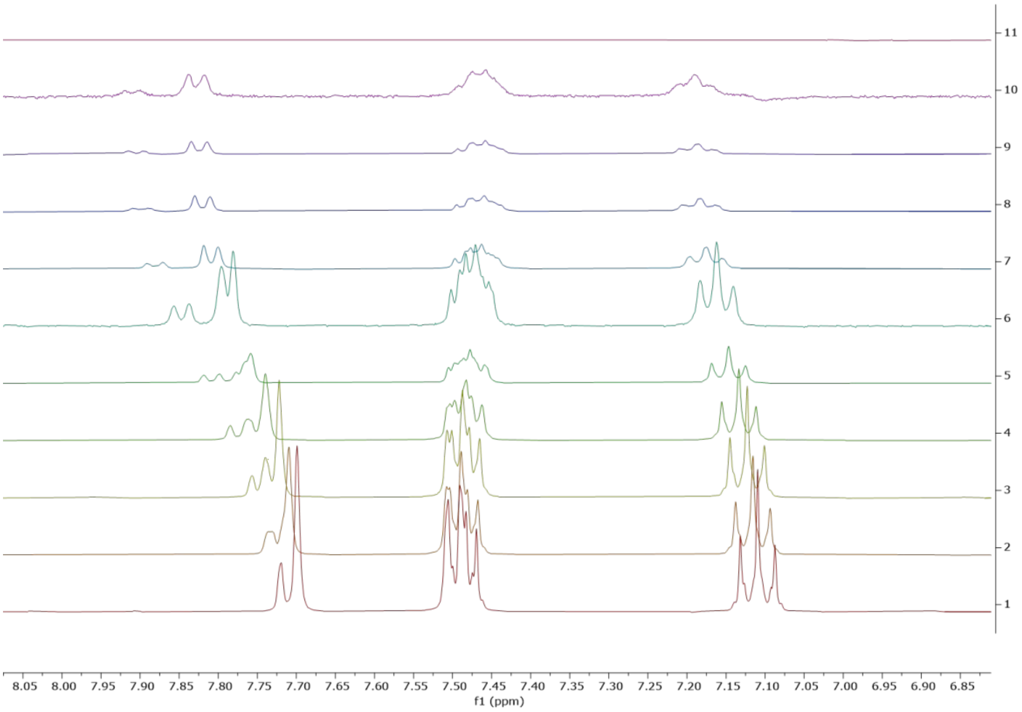

Figure S28. ^{1}H NMR spectra of CIT–SBX samples recorded for Job's plot analysis (aromatic region). The samples were prepared in 30 mM NaH_2PO_4 (10% D_2O), with the pH adjusted to 7.4 using 1 M NaOH. Spectra were recorded at 400 MHz using MeOH as an internal reference (δ = 3.310 ppm).

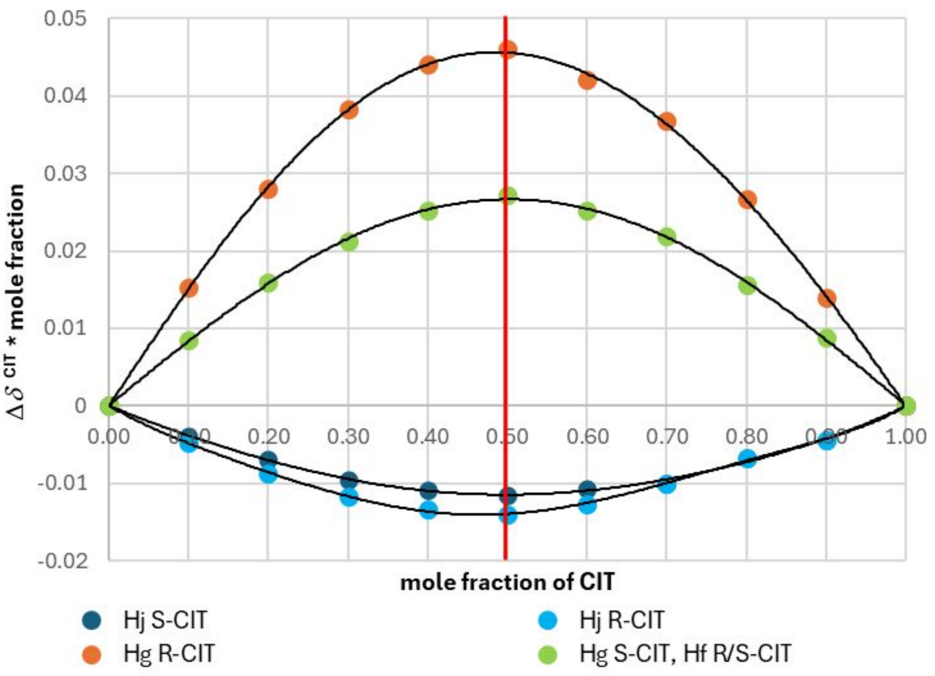

Figure S29. Job's plot of CIT-β-CD system, based on NMR chemical shift changes of the CIT aromatic region. Based on the results, the 1:1 host and guest ratio is proven by NMR.

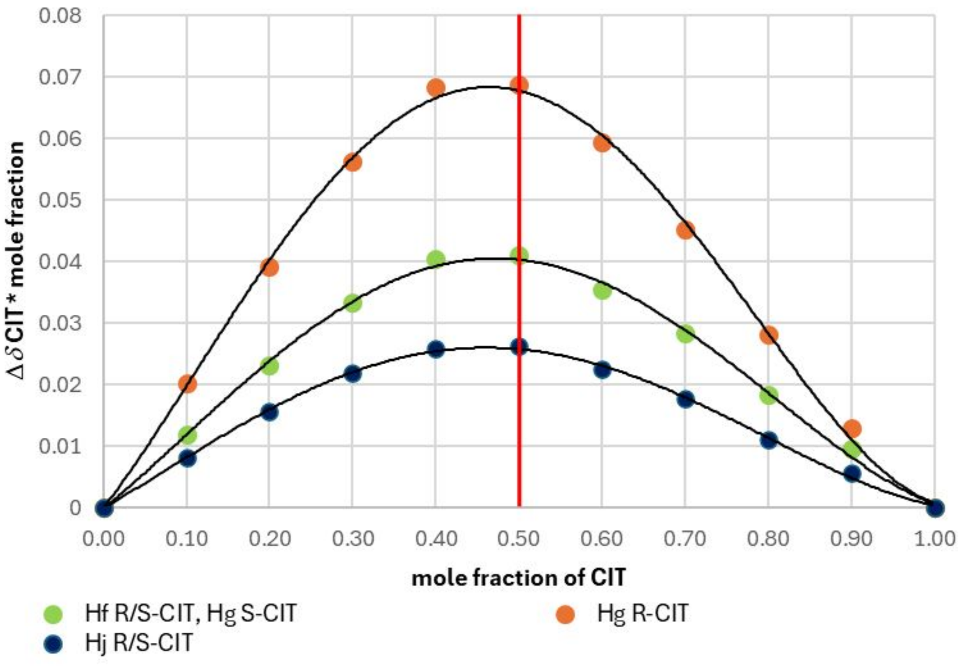

Figure S30. Job's plot of CIT-SBX system, based on NMR chemical shift changes of the CIT aromatic region. Based on the results, the 1:1 host and guest ratio is proven by NMR.

6.4 NMR titration method

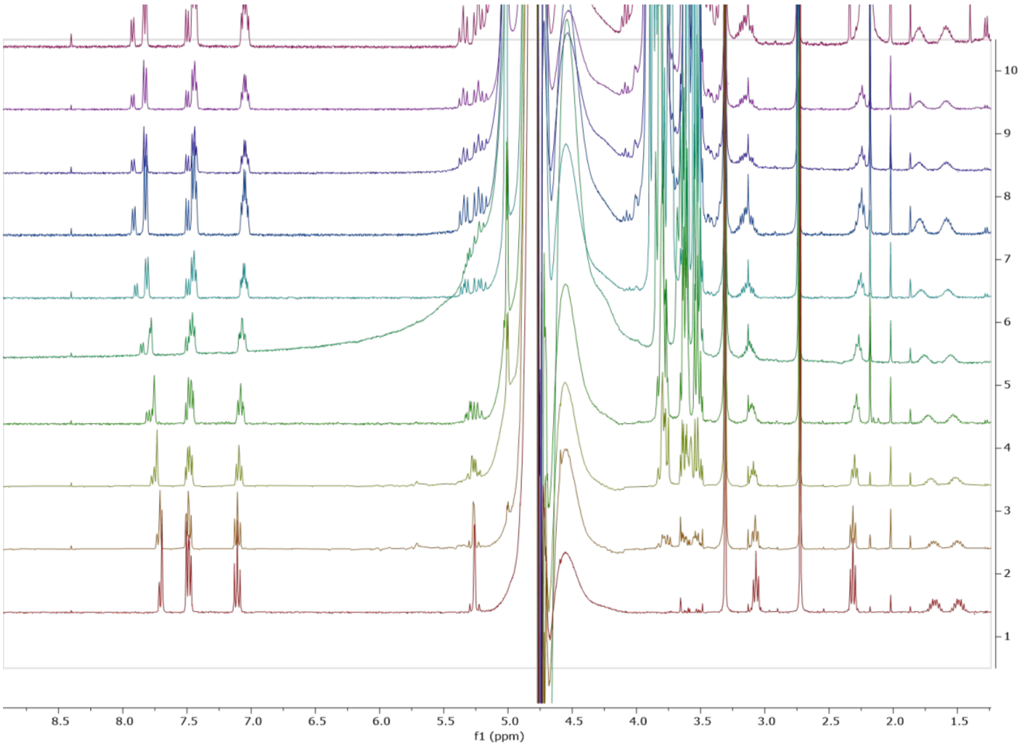

Figure S31. ^{1}H NMR spectra of CIT recorded during titration with β-CD (full spectra). The samples were prepared in 30 mM NaH_2PO_4 (10% D_2O), with the pH adjusted to 7.4 using 1 M NaOH. Spectra were recorded at 400 MHz using MeOH as an internal reference (δ = 3.310 ppm).

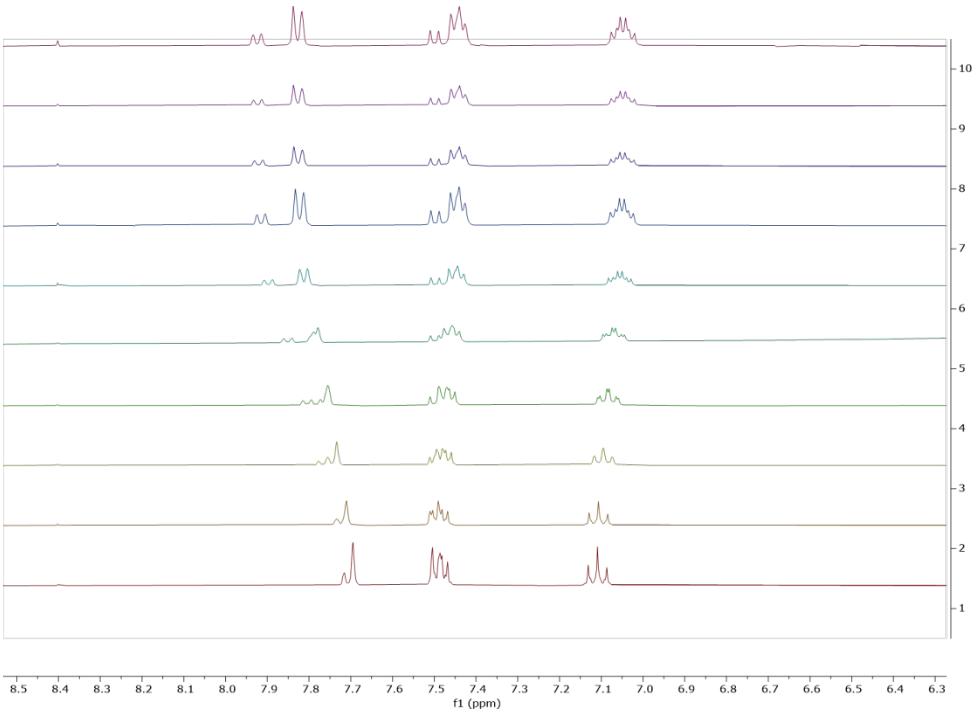

Figure S32. ^{1}H NMR spectra of CIT recorded during titration with β-CD (aromatic region). The samples were prepared in 30 mM NaH_2PO_4 (10% D_2O), with the pH adjusted to 7.4 using 1 M NaOH. Spectra were recorded at 400 MHz using MeOH as an internal reference (δ = 3.310 ppm).

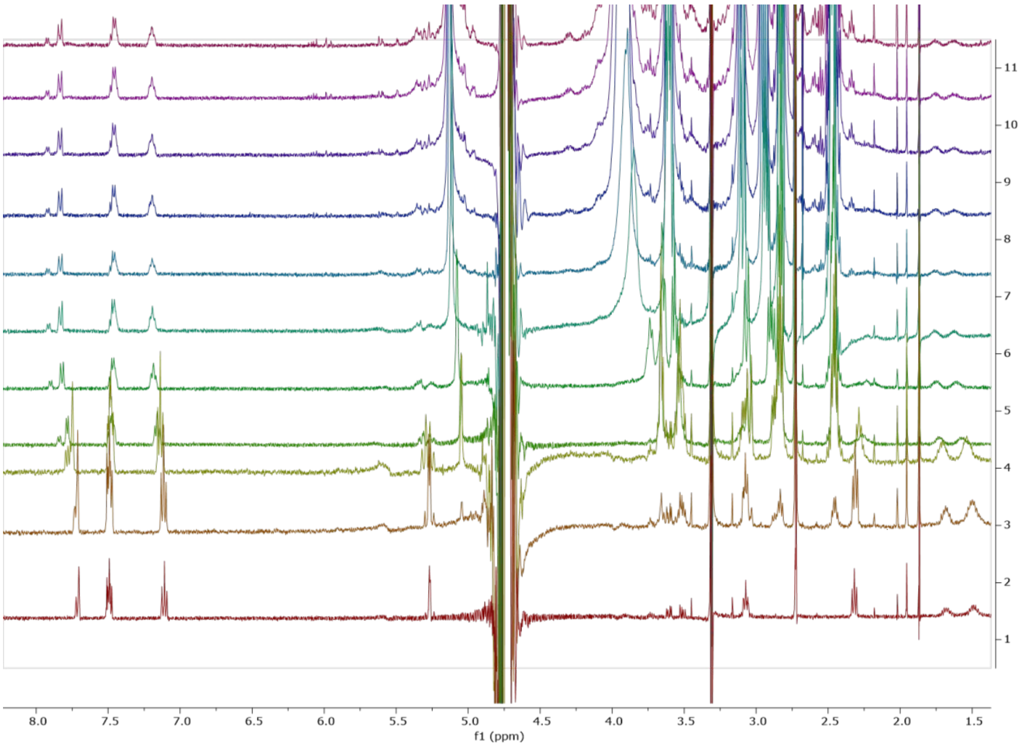

Figure S33. ^{1}H NMR spectra of CIT recorded during titration with SBX (full spectra). The samples were prepared in 30 mM NaH_2PO_4 (10% D_2O), with the pH adjusted to 7.4 using 1 M NaOH. Spectra were recorded at 400 MHz using MeOH as an internal reference (δ = 3.310 ppm).

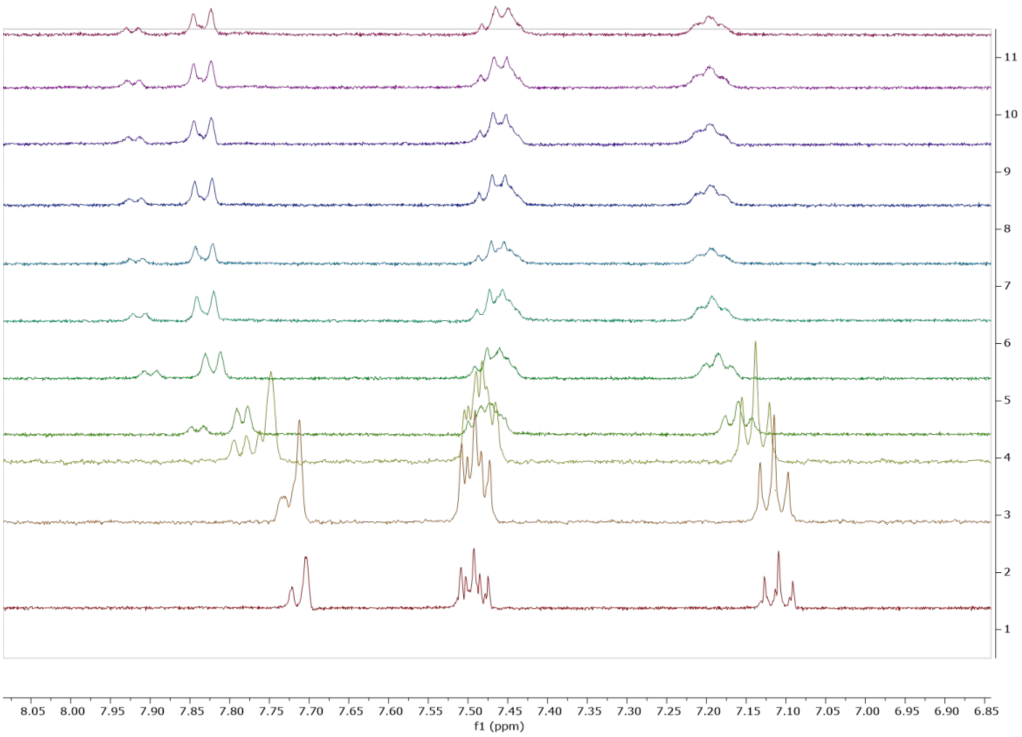

Figure S34. ^{1}H NMR spectra of CIT recorded during titration with SBX (CIT aromatic region). The samples were prepared in 30 mM NaH_2PO_4 (10% D_2O), with the pH adjusted to 7.4 using 1 M NaOH. Spectra were recorded at 400 MHz using MeOH as an internal reference (δ = 3.310 ppm).

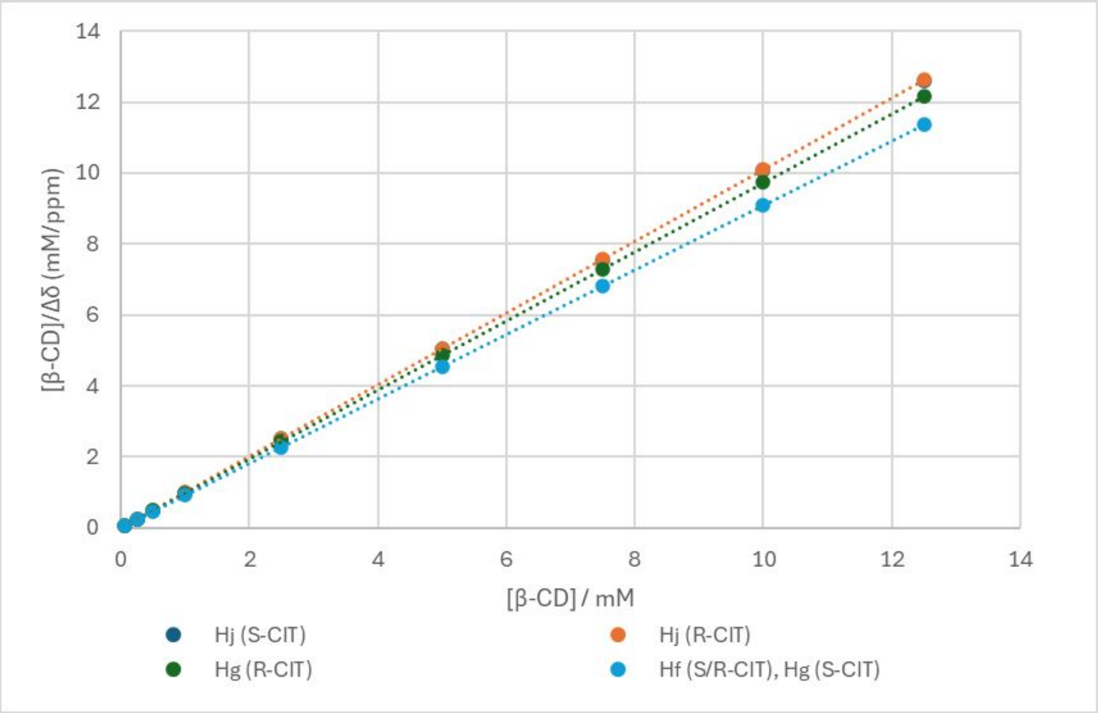

Figure S35. Scott plot of CIT- β-CD system. CIT-β-CD inclusion complex showing 1 : 1 stoichiometry.

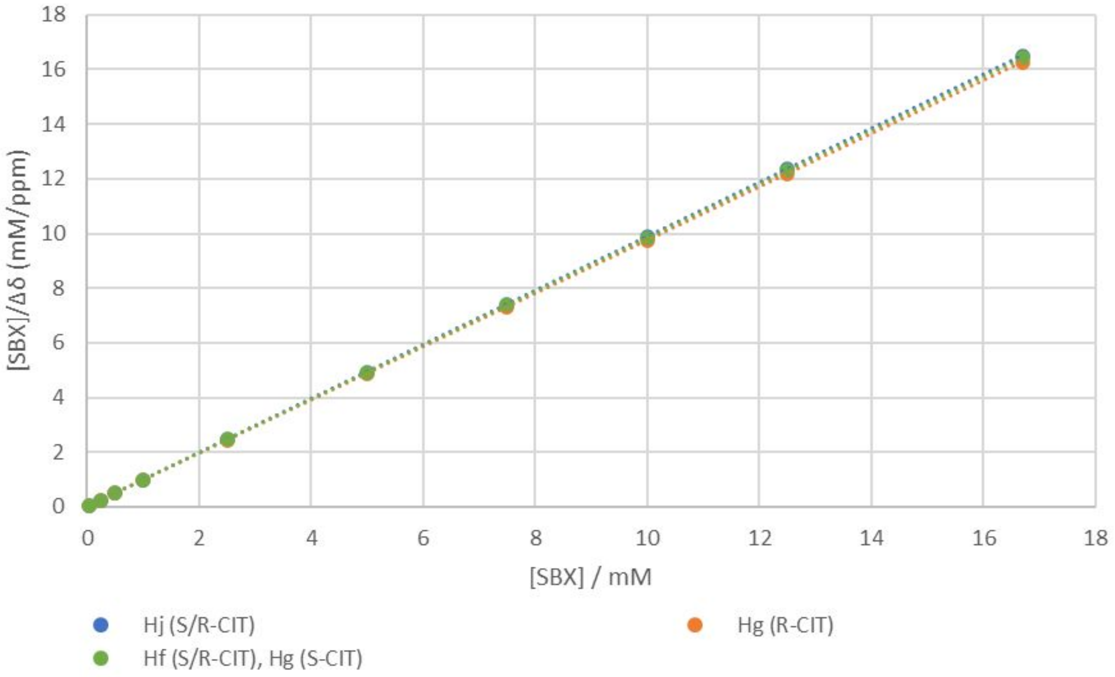

Figure S36. Scott plot of CIT-SBX system. CIT-SBX inclusion complex showing 1 : 1 stoichiometry.

6.5 1D ROESY NMR study

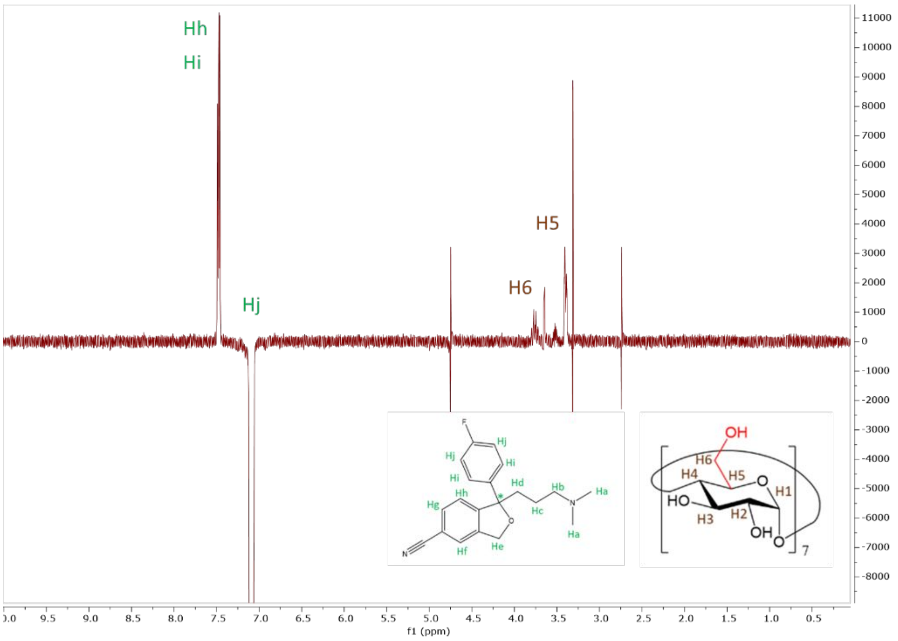

Figure S37. 1D ROESY NMR spectrum of the CIT–β-CD complex with selective excitation of the Hj proton of CIT (the excited proton appears with negative phase, while protons in intramolecular spatial proximity appear with positive phase). Measured at 500 MHz NMR using MeOH as an internal reference (δ = 3.310 ppm), in 30 mM NaH_2PO_4 (D_2O), with the pH adjusted to 7.4 using 1 M NaOD.

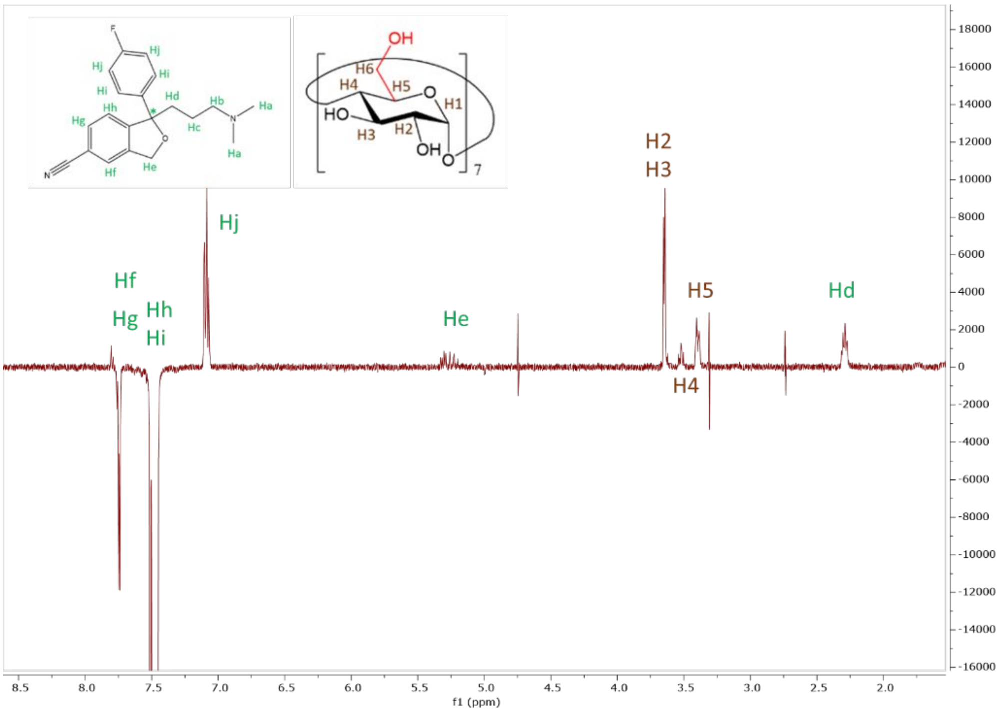

Figure S38. 1D ROESY NMR spectrum of the CIT–β-CD complex with selective excitation of the Hh-Hi protons of CIT (the excited proton appears with negative phase, while protons in intramolecular spatial proximity appear with positive phase). Measured at 500 MHz NMR using MeOH as an internal reference (δ = 3.310 ppm), in 30 mM NaH_2PO_4 (D_2O), with the pH adjusted to 7.4 using 1 M NaOD.

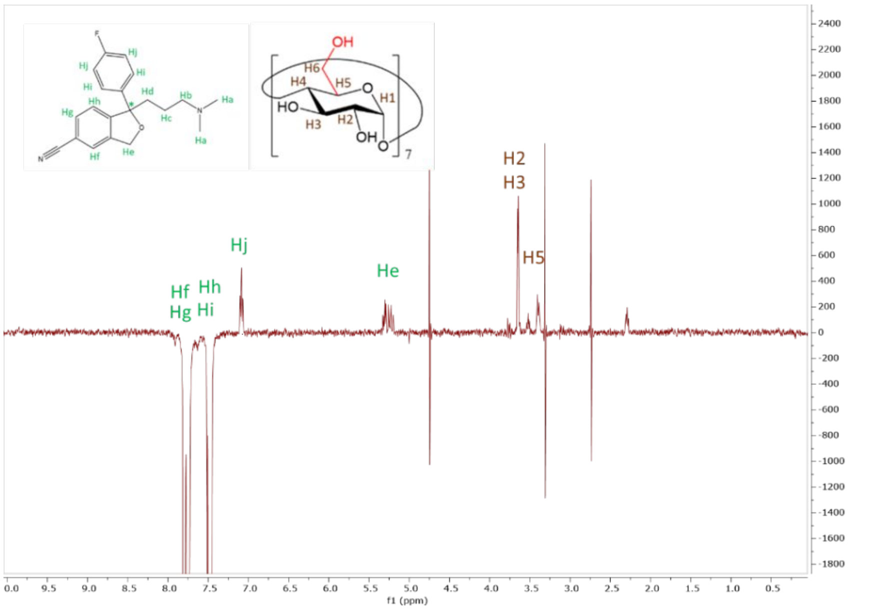

Figure S39. 1D ROESY NMR spectrum of the CIT–β-CD complex with selective excitation of the Hf-Hg protons of CIT (the excited proton appears with negative phase, while protons in intramolecular spatial proximity appear with positive phase). Measured at 500 MHz NMR using MeOH as an internal reference (δ = 3.310 ppm), in 30 mM NaH_2PO_4 (D_2O), with the pH adjusted to 7.4 using 1 M NaOD.

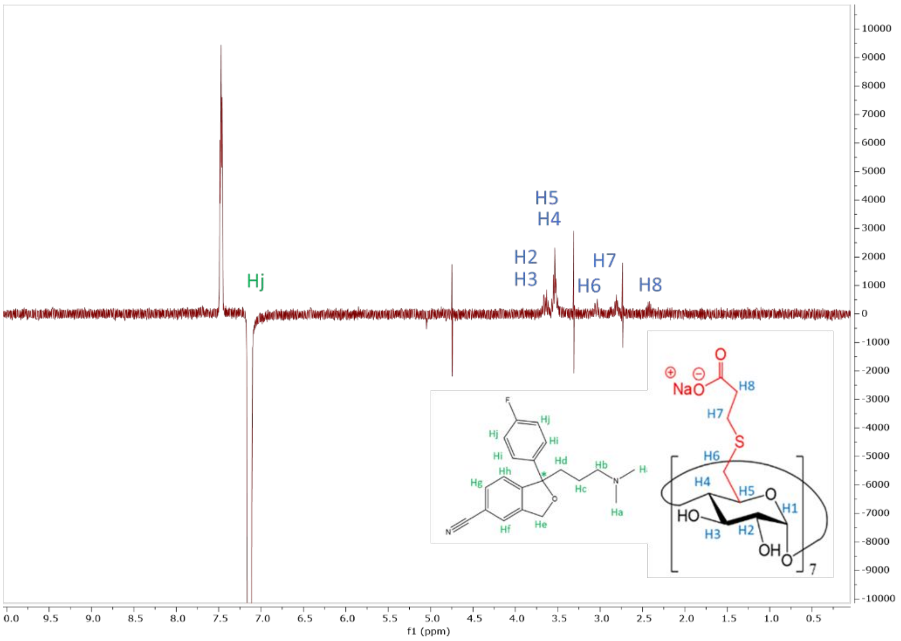

Figure S40. 1D ROESY NMR spectrum of the CIT–SBX complex with selective excitation of the Hj protons of CIT (the excited proton appears with negative phase, while protons in intramolecular spatial proximity appear with positive phase). Measured at 500 MHz NMR using MeOH as an internal reference (δ = 3.310 ppm), in 30 mM NaH_2PO_4 (D_2O), with the pH adjusted to 7.4 using 1 M NaOD.

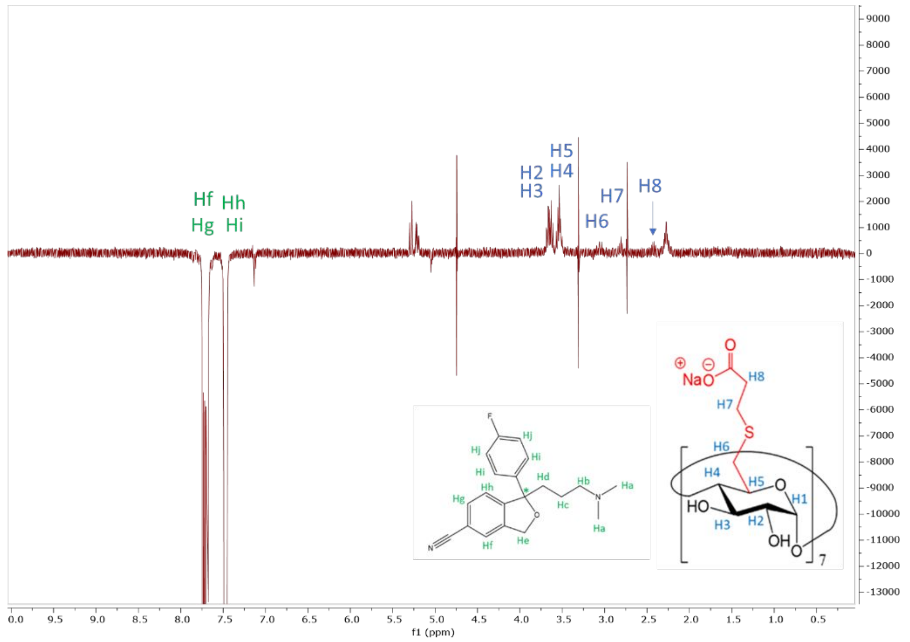

Figure S41. 1D ROESY NMR spectrum of the CIT–SBX complex with selective excitation of the Hf-Hg and Hh-Hi protons of CIT (the excited proton appears with negative phase, while protons in intramolecular spatial proximity appear with positive phase). Measured at 500 MHz NMR using MeOH as an internal reference (δ = 3.310 ppm), in 30 mM NaH_2PO_4 (D_2O), with the pH adjusted to 7.4 using 1 M NaOD.

7 References

(1) Popr, M.; Hybelbauerová, S.; Jindřich, J. A Complete Series of 6-Deoxy-Monosubstituted Tetraalkylammonium Derivatives of α-, β-, and γ-Cyclodextrin with 1, 2, and 3 Permanent Positive Charges. *Beilstein J. Org. Chem.* **2014**, 10, 1390–1396.

(2) Ashton, P. R.; Königer, R.; Stoddart, J. F.; Alker, D.; Harding, V. D. Amino Acid Derivatives of Cyclodextrin. *J. Org. Chem.* **1996**, 61 (3), 903–908.
